# Agriculture and Water Availability Show Contrasting Effects on Bats in a Mediterranean Island of Outstanding Chiropteran Biogeographical Value

**DOI:** 10.1002/ece3.70717

**Published:** 2024-12-23

**Authors:** Luca Cistrone, Ana Margarida Augusto, Gaetano Fichera, Hugo Rebelo, Danilo Russo

**Affiliations:** ^1^ Laboratory of Animal Ecology and Evolution (AnEcoEvo), Dipartimento di Agraria Università Degli Studi di Napoli Federico II Napoli Italy; ^2^ ICS, Instituto de Ciências Sociais Universidade de Lisboa Lisboa Portugal; ^3^ Department of Biogeography Trier University Trier Germany; ^4^ Naturkundemuseum Erfurt Erfurt Germany; ^5^ Departamento de Biologia Animal, Faculdade de Ciências, cE3c – Centre for Ecology, Evolution and Environmental Changes & CHANGE – Global Change and Sustainability Institute Universidade de Lisboa Lisboa Portugal

**Keywords:** bats, island, Mediterranean, pesticides, vineyards, water resources

## Abstract

With their unique ecosystems and evolutionary dynamics, small islands offer fascinating contexts to explore animal diversity. Island bats are key players in maintaining ecological balance. However, their populations are threatened worldwide, necessitating comprehensive research and conservation strategies. Pantelleria, a small Mediterranean island and a biogeographic crossroad between Europe and Africa, offers an excellent model to exemplify the challenges to bat conservation in such geographic contexts. We tested three hypotheses: (1) bats would show weak preference patterns for landscape composition due to the island's heterogeneous landscapes, (2) farmland (especially vineyards) would strongly affect bat activity, and (3) distance from water sources would highly influence bat richness and activity. We surveyed bats acoustically using Audiomoth recorders covering most of the island's surface. We recorded seven bat species, including endangered *Plecotus gaisleri*, *Myotis punicus* and *Rhinolophus mehelyi* . Bats showed weak preferences for specific landscape composition, but the dominant species ( *Pipistrellus kuhlii* ) decreased its activity for increasing portions of vineyards within the landscape. Moreover, distance to water critically influenced bat richness and activity. Agricultural expansion, pesticide use and human activities pose significant threats to bats on Pantelleria. We advocate for sustainable farming practices and careful water resource management to safeguard bat habitats and mitigate these threats. Conservation should target vineyards, a key economic resource to produce world‐renowned wine ‘Passito di Pantelleria’ by reducing pesticide use and adopting organic management. Water might be supplemented in critical dry habitats. We urge the preservation of bat diversity to support ecosystem health and resilience on small islands like Pantelleria.

## Graphical abstract

Our study reveals that while bats on Pantelleria exhibit weak preferences for specific landscapes, the expansion of vineyards significantly reduces the activity of the dominant species, *Pipistrellus kuhlii* , highlighting a key conflict between agriculture and bat conservation. We found that proximity to water sources is critical for bat richness and activity, emphasising the need for targeted water management strategies to support bat populations in this unique Mediterranean island ecosystem. To safeguard Pantelleria's bat diversity, we advocate for sustainable farming practices, particularly in vineyards and strategic water supplementation in dry habitats, ensuring the island's ecological balance and resilience.

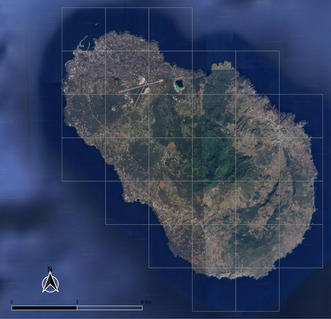

## 1 Introduction

Despite their diminutive size on the world map, islands, however small, often hold an outsized value for biodiversity (Russell and Kueffer 2019). These unique geographic systems, often considered natural evolutionary laboratories, harbour a disproportionate number of endemic species playing a crucial role in global biodiversity (Johnson and Stattersfield 1990; Kier et al. 2009; Caujape‐Castells et al. 2010). However, the biological uniqueness of many islands faces escalating threats due to human pressures (Fernández‐Palacios et al. 2021), making the study and conservation of island ecosystems imperative for the preservation of their biodiversity (Lagabrielle et al. 2009; Jaisankar, Velmurugan, and Sivaperuman 2018).

Among the diverse array of island‐dwelling organisms, bats emerge as key players in maintaining ecological balance (Jones et al. 2009). Bats provide vital ecosystem services, including pollination (Ratto et al. 2018), seed dispersal (Saldaña‐Vázquez et al. 2019) in tropical regions, and, globally, insect suppression (Russo, Bosso, and Ancillotto 2018; Tuneu‐Corral et al. 2023; Augusto et al. 2024), all of which underscore their ecological importance. Despite their critical role, bat populations worldwide face increasing threats, with many species experiencing declines and some teetering on the brink of extinction (Frick, Kingston, and Flanders 2020). Recognising the importance of understanding and mitigating these threats, global efforts are underway to unravel the complexity of bat roosting, foraging and movement ecology and implement effective conservation strategies (Berthinussen, Richardson, and Altringham 2014). The significance of islands for bats is accentuated by the fact that, in many instances, bats are the sole representatives of native mammals on these isolated land masses because their active flight makes island colonisation less challenging than for non‐flying mammals (Conenna et al. 2017).

On the other hand, the geographic barrier formed by expanses of sea contributes to the development of genetically distinct bat populations on islands, fostering the evolution of unique traits (McNab 2009). As a result, islands become pivotal arenas for bats' evolutionary history and the origin of endemic species (Conenna et al. 2017). For this reason, the taxonomic status of certain bat species on islands remains uncertain, underscoring the complexity of their evolutionary paths. Isolation may result in subtle but significant variations among populations, ultimately leading to distinct lineages and species (Loureiro, Engstrom, and Lim 2020). Recent advances in molecular analysis provide a powerful approach to unravel this taxonomic complexity, promising to refine our understanding of island bat diversity and possibly unveil previously unknown Evolutionary Significant Units (Teixeira, Smeraldo, and Russo 2023) and endemic cryptic species (Puechmaille et al. 2023), further enriching our comprehension of the intricate biogeographical relationships between bats and their insular systems.

In island climates prone to catastrophic events such as large wildfires, as typical of the Mediterranean (Ancillotto et al. 2021), the destruction of natural habitats poses a direct threat to bat species dependent on these environments, potentially leading to their irreversible loss (Conenna et al. 2017), a risk exacerbated by climate change. Invasive species, such as domestic cats, are also a major threat (Soto et al. 2023). Besides, human encroachment, particularly driven by tourism, poses a significant risk to island biodiversity (Steibl, Franke, and Laforsch 2021). Moreover, the distance from the mainland introduces a challenge for species occurring on both the mainland and an island, reducing gene flow and decreasing heterozygosity in island populations (Schmitt, Kitchener, and How 1995). Even with the capability of flying, distance remains therefore a significant challenge. Adverse events, such as habitat destruction or population decline, can still impede the recruitment of new individuals from the mainland, heightening the vulnerability of island populations. Additionally, islands typically have limited trophic and spatial resources, making any external pressure particularly impactful on inherently small populations (Frick, Hayes, and Heady 2008).

While bats on large islands such as Crete (Georgiakakis et al. 2010), Sicily (Di Salvo, Russo, and Sarà 2009) and Sardinia (Russo et al. 2014; Ancillotto et al. 2021) are relatively well‐studied, small islands often exhibit biogeographical and ecological knowledge gaps, complicating the development of adequate conservation plans (Georgiakakis et al. 2023). Pantelleria, situated respectively ca. 65 and 110 km away from the Tunisian and Italian (Sicilian) coasts, is notable for its high isolation. Its unique position as a crossroad between Europe and Africa contributes to its remarkable ecological richness (Antinucci et al. 2023), serving as a critical stopover for migratory wildlife and including several endemic species, or species belonging to African wildlife (Corso, Penna, et al. 2012; Muscarella and Baragona 2017; Massa et al. 2022; Sciandra et al. 2022). The bats' presence and ecological requirements on countless small islands such as Pantelleria are often under‐documented, leading to biogeographical knowledge deficits and a lack of suitable conservation strategies (Conenna et al. 2017).

Existing data on Pantelleria's bats are limited, with only three species listed in old studies (Felten and Storch 1970; Zava and Lo Valvo 1990). More recent work (Ancillotto et al. 2020; Fichera et al. 2022) has identified the presence of a bat species with an African distribution, *Plecotus gaisleri*, found, in Europe, exclusively on the Island of Pantelleria and in Malta. Pantelleria is not immune to the pervasive challenges faced by islands globally. Recurrent wildfires, encroachment of agriculture (especially for wine production) into natural habitats, and tourism development threaten the island's delicate ecological balances.

The characteristic landscapes of Mediterranean islands are highly heterogeneous and dynamic, forged by intricate interactions between ecosystem processes and human action, endowing them with unique natural, cultural and aesthetic values (Tzanopoulos and Vogiatzakis 2011). Pantelleria is no exception to this pattern: its landscapes are highly patchy and include scrubland, cultivated areas, human settlements and bare rock. The small size of habitat patches, coupled with the typically high mobility of bats (Voigt et al. 2017), raises questions about the possibility of different bat species associating with specific habitat types. However, agricultural habitats such as vineyards are known to support bat activity in the Mediterranean (Froidevaux, Louboutin, and Jones 2017; Baroja et al. 2021; Charbonnier et al. 2021) and benefit from the associated ecosystem service, so this cultivation type, widespread on the island, might play an important role for bats. Moreover, island size is the limiting factor for the presence of surface water resources (Viola et al. 2014). Hence, the scarcity of water typical of small Mediterranean islands, including Pantelleria, makes the few existing bodies of water overwhelmingly important for wildlife (e.g., Hadjikyriakou, Rogers, and Kirschel 2022). Water is of crucial importance for bats since they dehydrate easily through their body surface, particularly the respiratory system and the large wing membranes, so they must drink often to replenish water loss (Korine et al. 2016). Aquatic habitats also host key insect prey populations and represent prime foraging habitats for bats (Salvarina 2016).

We adopt Pantelleria as a typical model of the thousands of small islands scattered across the Mediterranean where bat ecology is unknown to test the following hypotheses:

1. The highly heterogeneous landscapes will imply weak habitat preference patterns in bats, so we predict little dependence on bat distribution and activity upon landscape composition.
2. Farmland (especially vineyards) constitutes a main land use type on Pantelleria, so we hypothesise that farmland will strongly influence bat activity and predict higher bat activity in vineyard‐dominated landscapes.
3. Given water scarcity, proximity to surface water is hypothesised to influence significantly both bat richness and activity. As such, we predict an increase in the number of bat species and the frequency of bat passes as the distance from water decreases.

This study aims to provide actionable insights for conservation planning on Pantelleria and comparable Mediterranean islands by identifying key spatial features and drivers of bat activity.

## 2 Material and Methods

### 2.1 Study Area

Pantelleria, situated within the Sicilian Channel, spans approximately 83 km^2^ and emerged around 320,000 years ago through volcanic activity (Mahood and Hildreth 1986). The island shows therefore significant hydrothermal features. Characterised by rugged terrain, it peaks at 834 m a.s.l., boasting a Mediterranean climate. The island's predominant vegetation includes Mediterranean shrubland and woodland patches, comprising oaks and indigenous conifers. Dominant agricultural activities include vineyards followed by capers and olive cultivation. Such cultivation types are often associated or interspersed so it is difficult to set boundaries in large‐scale analyses. Surface water is practically non‐existent in Pantelleria, except for minimal sources such as swimming pools, as water storage for human use has been a prime challenge ever since the Punic and Roman times (Mantellini 2015). A large volcanic lake gives the only significant exception, Bagno dell'Acqua, an endorheic, alkaline lake located in a volcanic caldera whose potential importance for bat foraging and drinking has been preliminarily suggested (Fichera et al. 2022).

### 2.2 Fieldwork

**FIGURE 1:**
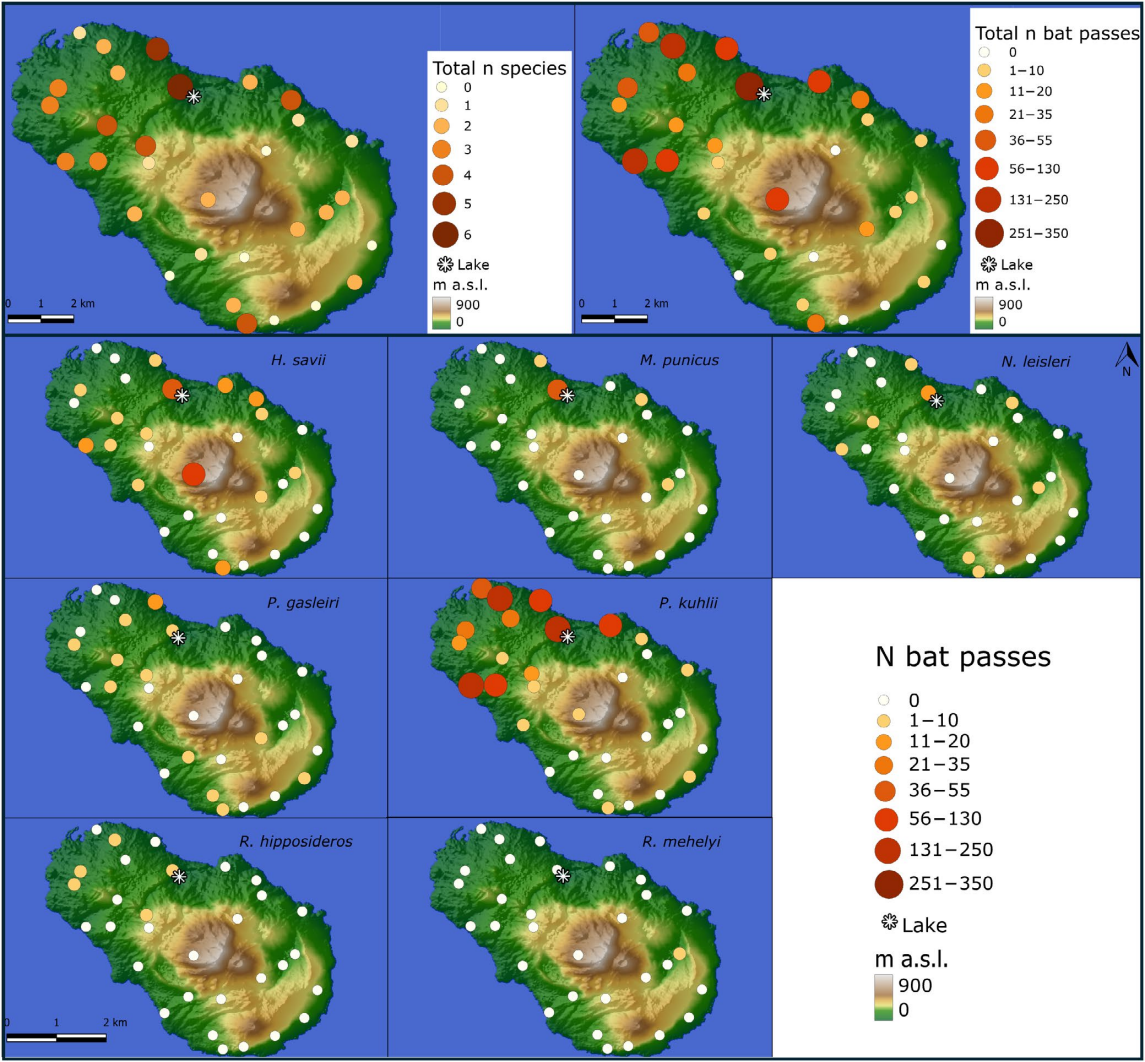
Location of sample points in Pantelleria where bat activity was recorded using passive automatic ultrasonic detectors. Both species richness and activity (total, and by species) are shown, with the diameter of the circles proportional to the recorded values, as illustrated in the legend. The asterisk denotes the lake position.

We recorded bat activity in May 2023. We primarily conducted the recordings by positioning Audiomoth recorders (https://www.openacousticdevices.info/audiomoth) at each of the 31 2‐km side quadrants into which we divided the island to ensure an even coverage of its territory (Figure 1; Figure S1). Such recorders have proved highly effective in previous surveys of bat activity on islands (Ferreira et al. 2022). Within each quadrant, the exact placement of each unit was randomly determined. The random generation of sampling points within each grid cell was performed using QuantumGIS software (QGIS Ver. 3.30, distributed under the GNU licence) via the ‘Random Points in Polygons’ tool. We established a minimum distance of 500 m between sampling points.

We deployed 5–10 AudioMoth detectors (Hill et al. 2018) each night at selected recording points across the study area, placing them inside waterproof cases provided by the manufacturer to ensure durability and protection from environmental conditions. These devices were mounted on poles, dry stone walls or rocks, positioned at heights of approximately 2 m from the ground. The orientation of each device was random, avoiding bias towards specific flight paths or landscape features. This setup minimised environmental obstructions and ensured a standardised approach across diverse habitats. Recordings were made continuously, with WAV files automatically split upon exceeding 4.3 GB in size. The devices remained operational for a day, starting half an hour after sunset and continuing until dawn, for approximately 8.5 h. The sampling rate set for the Audiomoth devices was 250 kHz. We avoided recording on rainy nights, and in all cases, the temperature was > 14°C.

We employed Wildlife Acoustics Inc.'s Kaleidoscope Pro ver. 5.6.3 software, to filter out files containing noise and to conduct a preliminary sorting of all recordings; however, all identifications were manually vetted, and no species identification was assumed to be correct without this additional verification. Kaleidoscope automatically divided the WAV files into 5‐s segments. We quantified bat activity using the measure of a ‘bat pass’, defined as a sequence of at least two pulses attributed to a single species within the 5‐s interval (Torrent et al. 2018). In Kaleidoscope, we included all species recorded on the island, except *Myotis punicus* and *Plecotus gaisleri*, which were unavailable in the software. Instead, we replaced these species with *Myotis myotis* , *Myotis blythii* and *Plecotus* spp. from Kaleidoscope's Europe library, assigning them to *M. punicus* and *P. gaisleri*, respectively, due to the similarity of their calls. We also included *Nyctalus leisleri* based on preliminary recordings that suggested its presence on the island. Neither feeding buzzes nor social calls were included in the analysis.

### 2.3 GIS and Statistical Analyses

To provide a multi‐scale analysis of habitat use, we established two buffer zones around each location whose radii were 100 m and 300 m. We avoided using larger buffer zones because the island's limited size would result in significant overlaps. Given this constraint, the number of recording sites selected (31) was sufficient to ensure comprehensive coverage while minimising the considerable overlap that would otherwise occur between the buffers used for landscape analysis.

For each sampling point, we analysed landscape composition to characterise land use in the surrounding areas at 100 m and 300 m scales. Land use classification was therefore performed considering a maximum radius of 300 m from the exact sampling point where the bat detector was placed. Identification and determination of different land use types were carried out through photointerpretation within a GIS environment, at a very detailed scale (1:1000 or 1:500), using freely available WMS services and QuantumGIS software (QGIS Ver. 3.30, distributed under GNU licence). To standardise the various land use types found, the fourth level Corine Land Cover classification was considered, allowing the identification of the following categories: Urban, Vineyard, Complex cultivation patterns (comprising smaller‐scale cultivation along with olive groves and caper cultivations), Bare rocks, Water bodies and Mediterranean vegetation. The latter was a mosaic habitat of Mediterranean macchia, garrigue and developing *Quercus ilex* woodland, with rare patches of fully developed woodland. For each buffer zone, besides quantifying the amount of each land use type, we also established tree cover density, altitude (both also extracted from Corine Land Cover) and distance to water (measured using QGIS tools between each point and Lago Bagno dell'Acqua). Subsequently, we analysed the influence of the environmental factors on bat richness and activity using the GLMM Hosmer–Lemeshow method, a widely employed statistical technique generating consistent and interpretable results (Hosmer et al. 2013). This method involves several sequential steps. We chose GLMMs with random effects at the sample point level, as recommended by Amorim et al. (2018). This approach allowed us to capture the full range of environmental variability by sampling multiple points within each habitat, rather than relying solely on fixed variables (land use types, as mentioned above), which could introduce bias from limited conditions. By incorporating random effects, we accounted for environmental variability not captured by our fixed variables. This ensured that comparisons between points within the same habitat were not confounded by unmeasured factors, providing a clearer understanding of how specific environmental factors influence bat richness and activity. The initial step involved standardising the data using the Z‐score function in R (Demircioğlu 2024). This process transforms the data to have a mean of 0 and a standard deviation of 1, thereby enhancing the precision and comparability of the results across different scales or distributions. Subsequently, single‐variable models were constructed for each response variable and each buffer zone (100 and 300 m). For each dataset analysed (Total number of species, Total number of passes, and numbers of passes of *P. kuhlii* and *H. savii* ), we initially performed a null model for each family (Poisson and negative binomial) to determine the most suitable family for the statistical tests. The model with the lowest Akaike Information Criterion (AIC) was selected for further analysis, as this criterion balances model fit and complexity, helping to identify the most appropriate model for the data (Richards 2005; Symonds and Moussalli 2011). Next, interactions between ecologically meaningful variables—such as the interaction between land use type and distance to water—were tested. Models were then constructed to include all variables found to be significant in single‐variable analyses, along with any significant interactions. In the final step, only variables and interactions that consistently remained significant were retained in the model. Across all models, the sample locations were included as a random effect to account for spatial dependency in the data.

We restricted the analysis to species richness, total number of bat passes and activity of the species for which a sufficiently high number of recordings were obtained. We performed the statistical analyses in RStudio (v.2022.07.2 Build 576 2009–2022 RStudio, PBC). To model the total number of bat passes and the number of passes of the species selected for analysis, we chose the negative binomial distribution due to the continuous and overdispersed nature of the data. For the number of bat species, we opted for the Poisson distribution, suitable for counting rare and discrete events. For both single‐variable and multiple‐variable models (GLMM), we used an analysis of variance to test the significance of the model employing the ‘glmmadmb’ function with the ‘nbinom’ and ‘poisson’ distributions from the ‘glmmADMB’ package (Fournier et al. 2012). We considered statistical significance at a *p* value < 0.05.

## 3 Results

We obtained 1390 recordings of bat echolocation sequences spanning seven bat species (Figure 1). Across almost all species, the highest activity levels were observed near the island's only lake. The only exception was *Hypsugo savii* , whose highest activity (77 bat passes) was recorded in the island's mountainous region. We recorded echolocation calls consistent with *Nyctalus leisleri* , with some calls displaying the typical alternation between frequency‐modulated (FM) and constant‐frequency (CF) components (Figure S2). Quantitative analysis was limited to the total number of species, total bat activity and the activity of only *Pipistrellus kuhlii* and *Hypsugo savii* , for which a sufficiently high number of recordings were obtained (Table S1). Full single‐ and multiple‐variable GLMM models are presented in Tables S2–S13, whereas only the final models are shown in the main text. For the remaining species, no statistical analysis was possible. However, a visual inspection of the number of bat passes suggested no specific pattern across the island, except for *Myotis punicus* , whose highest activity was recorded in the lake area (Figure 1).

**FIGURE 2:**
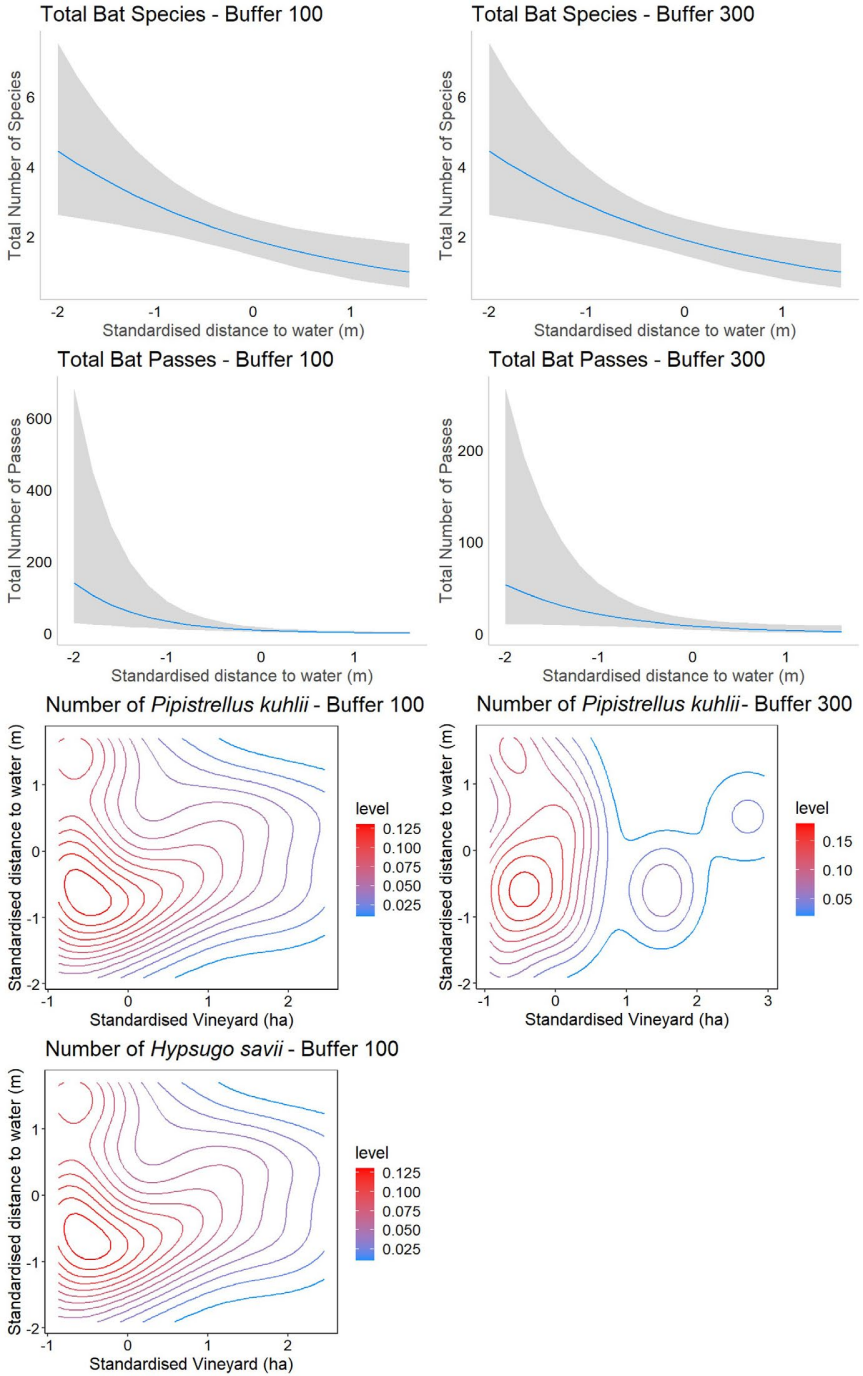
Predicted effects of final models within 100 m (left graph) and 300 m (right graph) buffers. All *Z*‐scores range from −3 to +3 standard deviations. The graphs depict the relationship between total bat species and number of passes with distance to water. Shaded areas indicate 95% confidence intervals. Additionally, predicted effects on *Pipistrellus kuhlii* number of passes in Vineyards and distance to water are shown, with contour lines representing equal pass regions. Similarly, the interaction between Vineyards and distance to water on *Hypsugo savii* number of passes within a 100 m buffer is displayed. Contour lines indicate predicted number of pass estimates whose values are expressed by the colour gradient ranges.

The distance from the lake was the sole factor influencing species richness and total bat activity, with lower values recorded at greater distances from its closest shores at both spatial scales (Table 1, Figure 2). The activity of *Pipistrellus kuhlii* decreased with increasing distances from water and the presence of vineyards surrounding the recording point, at both spatial scales considered (Table 1, Figure 2). In the case of *H. savii* , only a borderline significant interaction occurring within 100 m buffers between the extent of vineyards and water was detected. Higher activity was also detected at sites closer to the lake shores for equal extents of vineyards within the 100 m buffer (Table 1; Figure 2). No effect was detected for this species at 300 m (Table S12).

**TABLE 1:** Relationship depicted by GLMMs multiple‐variable model between the total number of bat species, total number of bat passes, *Pipistrellus kuhlii* and *Hypsugo savii* passes, and the final explanatory variables, for 100 m and 300 m buffers. Significant *p* values are highlighted in bold.

Coefficients	Estimate	Std. error	*z* value	*p*	*R* ^2^ (fixed effects)	*R* ^2^ (fixed + random effects)	AIC
Total species – buffer 100 m							
(Intercept)	0.642	0.136	4.73	**2.2e‐06**	0.306	0.306	107.6
Distance to water	−0.424	0.140	−3.04	**0.002**			
Total species – buffer 300 m							
(Intercept)	0.642	0.136	4.73	**2.2e‐06**	0.306	0.306	107.6
Distance to water	−0.424	0.140	−3.04	**0.002**			
Total passes – buffer 100 m							
(Intercept)	2.088	0.361	5.78	**7.3e‐09**	0.375	0.978	268.3
Distance to water	−1.426	0.371	−3.85	**1.2e‐04**			
Total passes – buffer 300 m							
(Intercept)	2.088	0.361	5.78	**7.3e‐09**	0.375	0.978	268.3
Distance to water	−1.426	0.371	−3.85	**1.2e‐04**			
*P. kuhlii* – buffer 100 m							
(Intercept)	0.774	0.489	1.58	0.113	0.585	0.963	212.2
Vineyards	−1.471	0.522	−2.82	**0.005**			
Distance to water	−2.015	0.452	−4.45	**8.5e‐06**			
*P. kuhlii* – buffer 300 m							
(Intercept)	0.790	0.482	1.64	0.102	0.598	0.963	212.7
Vineyards	−1.516	0.564	−2.69	**0.007**			
Distance to water	−1.960	0.441	−4.44	**9.0e‐06**			
*H. savii –* buffer 100 m							
(Intercept)	−0.344	0.589	−0.58	0.560	0.270	0.870	154.0
Vineyards	−0.434	0.462	−0.94	0.350			
Distance to water	−0.376	0.593	−0.63	0.530			
Vineyard: Distance to water	1.437	0.700	2.05	**0.040**			

## 4 Discussion

### 4.1 Bat Species Assemblage

Our study confirmed the presence of the six bat species previously found on Pantelleria Island (Ancillotto et al. 2021; Fichera et al. 2022), including some species of outstanding biogeographic and conservation value such as *P. gaisleri*, *M. punicus* and *R. mehelyi* . Besides, we provide the first potential record of Leisler's bat ( *Nyctalus leisleri* ) for the island, where it appears to be infrequent. Caution is warranted as our study relied solely on call identification without physical captures. We cannot fully exclude the possibility that our recordings could be confounded with calls from other species with similar calls, such as, for example, other bats in the genus *Nyctalus* or *Eptesicus*. However, the alternation between constant frequency (CF) and frequency‐modulated (FM) echolocation calls observed in some recordings, characteristic of the genus *Nyctalus*, and the frequencies of maximum energy falling within the species' typical range are consistent with our identification. *Nyctalus leisleri* is known to be a long‐distance, sex‐biased migrant capable of crossing the open sea (Ahlén, Baagøe, and Bach 2009), and it is found in both Europe and the Maghreb. Therefore, migratory routes may connect the island with these regions, suggesting that the Pantelleria population might not be resident but rather a transient or migratory presence. *Rhinolophus mehelyi* shows a highly precarious status across Europe, being assessed as Endangered, with declining populations in much of its range and close to extinction in Sicily (Russo and Cistrone 2023a). European populations of *P. gaisleri*, restricted to Malta and Pantelleria, are likewise endangered and declining under the pressure of human action, especially wildfires and residential and commercial development (Russo and Cistrone 2023b). *Myotis punicus* is now assessed as Vulnerable in Europe (Russo and Cistrone 2023c): its situation in the nearby Sicily, where the species was only recently reported (Bogdanowicz et al. 2015), is practically unknown, while in Malta, over half of the population disappeared in the 1980s‐1990s and would now be stable at 400–450 bats (Borg 2002; Russo and Cistrone 2023c). *Rhinolophus hipposideros* on Pantelleria is relatively widespread yet with low densities and highly threatened by the renovation of traditional buildings (‘dammusi’) where small nursery colonies can be found (Fichera et al. 2022). Its echolocation calls show an especially high peak frequency (Fichera et al. 2022), suggesting a peculiar genetic identity of this insular population which warrants further investigation. These data alone highlight the outstanding conservation value of Pantelleria for bat species that are in several cases at high risk across much of their range.

A recent PhD dissertation (Massaad 2024) reported for Pantelleria *P. kuhlii* , *H. savii* and *R. hipposideros* also detected in our study, as well as *P. pipistrellus* and *Tadarida teniotis* . Although the presence of these two species cannot be ruled out, we argue that some *P. kuhlii* calls might be misclassified. In simplified bat assemblages, pipistrelle echolocation calls may ‘invade’ acoustic species of other bats from the same genus when they are not sympatric (Montauban et al. 2021). As for *T. teniotis* , we did not record the species on the island, not only through the automatic detectors deployed for this study but also after repeated car transects and opportunistic recordings made using hand‐held bat detectors in June and September 2023. Likewise, Fichera et al. (2022) failed to detect the species. However, this bat flies fast and high (O'Mara et al. 2021) and is quite common on Sicilian islands (e.g., Fiore, Violani, and Zava 1992; Zava, Violani, and Mannino 1994), where it often hunts over the sea (D. Russo, personal observation). The species is unmistakable to identify from its echolocation calls, audible and so detectable by the unaided ear (Russo and Jones 2002), which makes it difficult to overlook. However, it might not be resident in Pantelleria and occurring only occasionally or in specific periods. Additional bat surveys are necessary to improve the accuracy of documenting species' presence on the island. Capturing bats is particularly challenging due to the scarcity of suitable habitats and the low numbers of bats throughout the island, highlighting the need for alternative methods such as incorporating captures, environmental DNA (eDNA), or guano sequencing to enhance species detection and documentation.

### 4.2 Bat Activity and Habitat Use

A notable aspect is the low bat activity recorded across the island, averaging 5.3 passes per hour of recording. Besides, in agreement with our hypothesis regarding the highly patchy nature of landscapes, bats show weak relationships with landscape composition. Overall, bats are rare on Pantelleria, although we might have underestimated the activity of species such as rhinolophids and *P. gaisleri*, emitting weak or directional echolocation calls and therefore potentially overlooked in acoustic surveys (Russo, Ancillotto, and Jones 2018). However, especially for *P. gaisleri* and *R. mehelyi* , roost surveys and mist‐netting conducted opportunistically on the island essentially confirmed this observation (D. Russo, personal observation).

Additionally, *M. punicus* , a species emitting strong, easy‐to‐detect echolocation calls, occurred across much of the island again with low activity levels, with the only exception at the lake area, where activity was significant. Nocturnal observations employing hand‐held detectors and night‐vision devices confirmed that the species often forages over the lake surface or along its shores (D. Russo, personal observation). The typical foraging habitats of *M. punicus* outside Pantelleria are semi‐open Mediterranean maquis, grasslands, pastures and forest edges, and occasionally orchards or vineyards, where the species typically gleans its prey from the substrate (Ruedi 2020). While we also observed *M. punicus* in maquis and vineyards, the high activity of the species over the lake resembles that of the more specialised ‘trawling *Myotis’* bats (Denzinger and Schnitzler 2013) present in other regions but not on Pantelleria. Pantelleria would, therefore, represent a case of ecological release (Herrmann, Stroud, and Losos 2021), where the species has expanded its niche in allopatry with water habitat specialist *Myotis* bats.

What causes the generally low activity of bats on Pantelleria? Food resources on small islands are typically limited (McNab 2002), which may reduce bat population sizes and activity levels. However, we propose that human action, including massive renovation for tourism development of long‐abandoned traditional buildings that represent important roosting sites (Fichera et al. 2022), and agricultural expansion, may also play a significant role.

Due to the limited number of passes recorded for most species, our quantitative analysis concentrated primarily on the overall bat activity and that of *P. kuhlii* and *H. savii* . Interestingly, in all such cases, bats displayed minimal preferences for landscape composition, consistent with our initial hypothesis. However, the observed generalist foraging habitats of *P. kuhlii* and *H. savii* (also making the bulk of the ‘total bat activity’) may contribute to their limited preferences. Nevertheless, at least on the mainland, these species still present habitat preference patterns, as radiotracking studies showed (Serangeli et al. 2012; Ancillotto et al. 2018).

Despite their generalist tendencies, a negative influence of vineyards on bat activity was detected, which rejected our initial hypothesis. This was especially evident for *P. kuhlii* , which was unexpected given its typical foraging behaviour in farmed landscapes, especially those managed organically (Ancillotto, Scaramella, et al. 2023). The high landscape diversity of Pantelleria would typically promote bat activity in vineyards, as observed elsewhere (Froidevaux, Louboutin, and Jones 2017; Rodríguez‐San Pedro et al. 2019), potentially benefiting grape production through natural pest control and reducing the need for pesticides (Charbonnier et al. 2021). However, the results contradicted the trend and our predicted hypothesis. We suggest that the response to vineyards may be influenced by the type of management adopted on the island. While some properties are managed organically, most are still under conventional management (A. Biddittu, pers. comm.), involving herbicides and insecticides, and removing natural vegetation from inter‐rows.

The significant use of pesticides and fertilisers on Pantelleria has been identified as a concern for sensitive animal species, such as dragonflies (Corso, Janni, et al. 2012). Synthetic pesticides are also employed to control the bagrada bug (*Bagrada hilaris*), an invasive hemipteran present only in Malta and Pantelleria, where it causes substantial damage to caper production (Mainardi et al. 2023). Pesticides can easily spread across adjoining cultivation types or reach natural habitats in Pantelleria's patchy landscapes. Removing natural vegetation from vineyard inter‐rows is also an action known to bring about adverse consequences for biodiversity (e.g., Winter et al. 2018; Zanettin et al. 2021), and, specifically, arthropods (e.g., Winter et al. 2018; Zanettin et al. 2021), which might reduce further the trophic resource available to bats in such ecosystems. Agriculture via the use of pesticides is suspected to negatively affect the activity of other insular bats, such as *Plecotus austriacus* in Madeira (Ferreira et al. 2022).

In agreement with our hypothesis, water, represented by the only lake on Pantelleria in our analysis, has indeed proven to be the primary factor influencing bat distribution and activity: species richness, total bat activity and the activity of *P. kuhlii* , all increased with shorter distances to water at both spatial scales considered. *Hypsugo savii* showed no preference for landscape composition; however, at 100 m, a weak but significant interaction between vineyards and distance to water was detected, indicating higher bat activity in landscapes where vineyards were present at longer distances from the lake. We argue that this effect arises from other environmental factors. Vineyards closer to the lake have been reported to be more severely treated with pesticides and synthetic fertilisers (Corso, Janni, et al. 2012; Corso, Penna, et al. 2012), which might explain why they are less attractive for this species, typically foraging in farmland on mainland Italy (Ancillotto et al. 2018; Ancillotto, Falanga, et al. 2023).

In our analysis, we could not consider the effect of the often‐strong wind on the island, which is difficult to record accurately as its intensity is highly variable over space and time. However, we suspect wind may considerably affect prey distribution and likely influence bat activity. Finally, given that our study focused on the responses of bats to landscape composition rather than temporal variations in activity, our primary objective was to maximise spatial coverage across the island's diverse habitats. While bat activity can fluctuate nightly, single‐night sampling at multiple sites still provides valuable insights into spatial patterns of activity and habitat associations. Although rare species may have been under‐sampled, our study aimed to identify broad habitat and landscape effects rather than provide exhaustive inventories of species diversity. However, more comprehensive sampling covering different periods of the year would be needed to assess any seasonal effects on bat activity.

### 4.3 Conservation Implications

Wine production in Pantelleria has gained significant prominence both in Italy and internationally within the modern Italian wine system, with two wines (Moscato di Pantelleria and its Passito di Pantelleria variants) being granted DOC status (Tudisca, Sgroi, and Testa 2011). In 2014, UNESCO included Pantelleria's planting of terraced head‐trained bush vines (‘vite ad alberello’) in the ‘Intangible Cultural Heritage of Humanity’, signifying not only great economic importance but also overwhelming cultural value. Encouraging practices such as organic farming, reducing pesticide use and promoting cover crops between rows could significantly enhance biodiversity. Future studies should address the potentially beneficial effects of maintaining grass cover in the inter‐rows of vineyards. These changes would safeguard bats and boost their pest control role (e.g., Tuneu‐Corral et al. 2023). However, structural improvements should be complemented by efforts to mitigate pesticide use, ensuring habitats designed to support bats do not inadvertently become ecological traps (Russo et al. 2024).

Water is a resource of prime importance for humans and wildlife in Mediterranean islands and requires careful management. Our study showed a decrease in bat richness, total activity and *P. kuhlii* activity as the distance from the nearest water source increased, highlighting the importance of water availability for bats. Therefore, it is logical to consider adding water sites as a conservation measure. Increasing the number of water reservoirs such as fire‐suppression ponds would be a win‐win initiative, enhancing safety against wildfires and providing valuable drinking and foraging sites for bats. However, we appreciate that water availability is a long‐standing issue on Mediterranean islands such as Pantelleria, so implementing this conservation measure might not be easy, at least on a large scale. Increasing the availability of drinking sites in key areas would, in principle, be of paramount value to bats (Korine et al. 2016), and future work might test its effectiveness using a before‐after‐control‐impact (BACI) design.

Additionally, given the key role of Lake Bagno dell'Acqua for foraging bats, its habitat quality warrants special attention and should be carefully preserved, with a particular focus on reducing pesticide use in the surrounding farmland. Other critical actions to protect bats include effectively preventing and combating wildfires, and strictly preserving the few remaining underground roosts and building roosts.

The unique circumstances of island ecosystems, characterised by stochastic colonisation and limited resources, can result in disharmonic communities (König et al. 2021) and minimal functional redundancy (McConkey and Drake 2015), as in Pantelleria. Therefore, even a single bat species may play a pivotal and irreplaceable role (Florens et al. 2017). The scarcity of resources and the chance‐like mechanisms of colonisation mean that the disappearance of a particular bat species may leave a void with no alternative species to undertake the same crucial ecological task (Jones et al. 2009; Conenna et al. 2017). This underscores the indispensable contribution of bats to maintaining islands' environmental balance and ecosystem health, and accentuates the importance of each species, necessitating targeted conservation measures (Conenna et al. 2017). Our study remarks the high value and fragility of bat communities on islands like Pantelleria, and we urge the development of specific Action Plans for Mediterranean island bats, involving the EU and IUCN directly, besides the relevant national authorities involved, to safeguard what remains of bat diversity in these irreplaceable geographic systems.

## Author Contributions

**Luca Cistrone:** conceptualization (equal), data curation (lead), formal analysis (lead), investigation (lead), methodology (equal), writing – original draft (lead), writing – review and editing (equal). **Ana Margarida Augusto:** data curation (equal), formal analysis (lead), investigation (equal), methodology (equal), validation (equal), writing – original draft (supporting), writing – review and editing (supporting). **Gaetano Fichera:** data curation (supporting), investigation (supporting), resources (supporting), validation (equal), writing – original draft (supporting), writing – review and editing (supporting). **Hugo Rebelo:** conceptualization (equal), formal analysis (equal), investigation (equal), methodology (equal), supervision (equal), validation (lead), writing – original draft (equal), writing – review and editing (equal). **Danilo Russo:** conceptualization (lead), data curation (equal), formal analysis (equal), funding acquisition (lead), investigation (lead), methodology (lead), project administration (lead), resources (lead), software (lead), supervision (lead), validation (equal), visualization (equal), writing – original draft (lead), writing – review and editing (lead).

## Conflicts of Interest

The authors declare no conflicts of interest.

## Supporting information


Appendix S1

## Data Availability

All data used in this study are part of the Supporting Information (Table S1).
