# Supplementary material for: Agriculture and Water Availability Show Contrasting Effects on Bats in a Mediterranean Island of Outstanding Chiropteran Biogeographical Value
Source: Ecol Evol. 2024 Dec 23;14(12):e70717. doi: 10.1002/ece3.70717 (PMC11664209; doi:10.1002/ece3.70717)
Supplement: Supplementary file 1 — Appendix S1 [file ECE3-14-e70717-s001.docx]

**Supplementary Material**

**
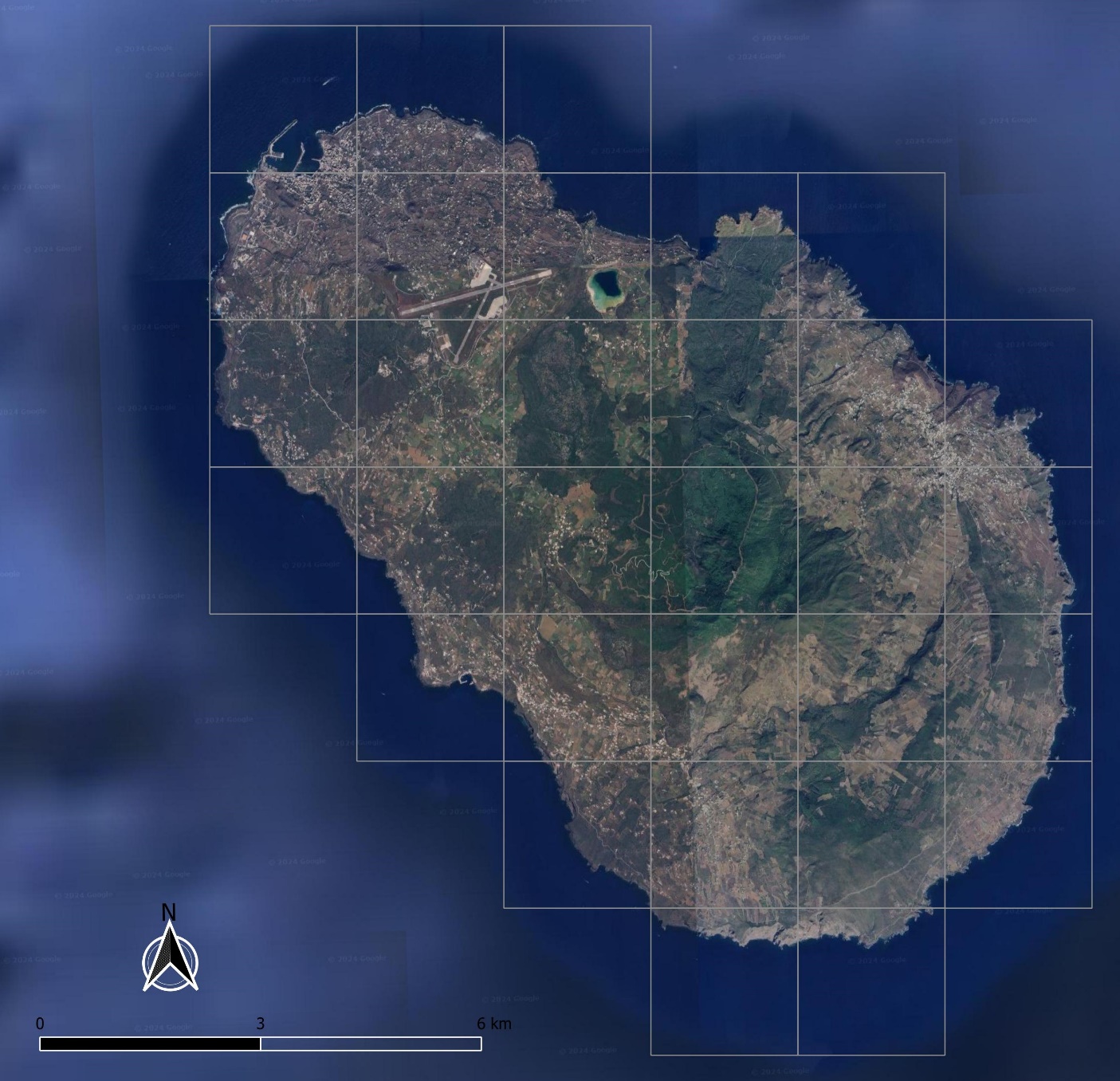
**

Figure S1. The 31 quadrants, each with a 2 km side length, into which we divided Pantelleria for bat surveys to ensure even coverage of the island. Map Data © [2024] Google Earth.


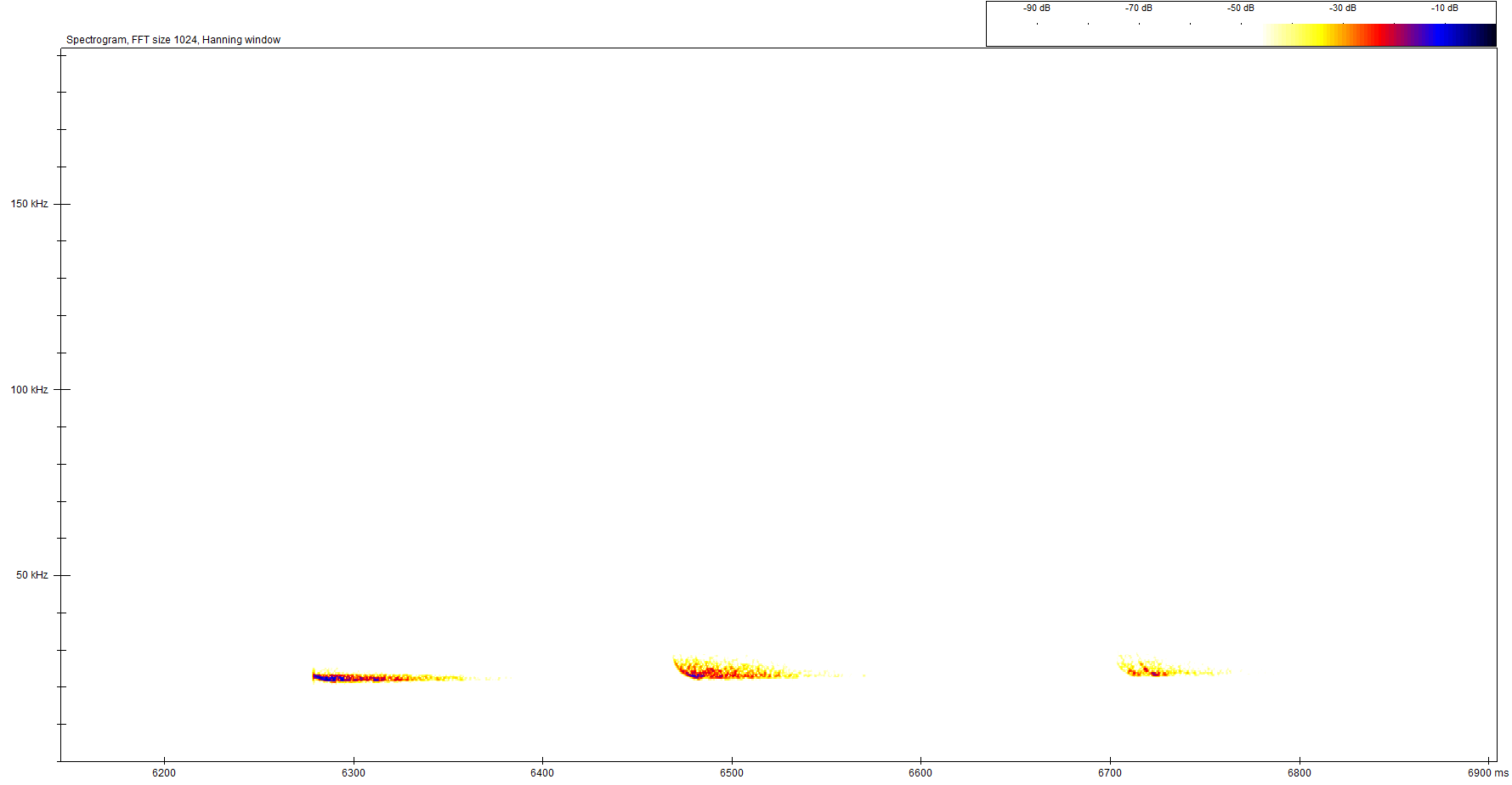


Figure S2. Echolocation calls consistent with *Nyctalus leisleri*. Calls alternate between frequency-modulated (FM) and constant-frequency (CF) structures. End frequencies between 21.4 kHz – 22.3 kHz.

Table S1 – Dataset used for statistical analysis. Lines correspond to grid cells and show landscape composition at 100 and 300 m radii.

| Buffer | Richness | Total n passes | *P. kuhlii* | *H. savii* | *M. punicus* | *N. leisleri* | *P. gaisleri* | *R. mehelyi* | *R. hipposideros* | Urban | Vineyard | Complex cultivation patterns | Bare  rocks | Mediterranean vegetation | TCD sum | TCD mean | Altitude | d_water |
| --- | --- | --- | --- | --- | --- | --- | --- | --- | --- | --- | --- | --- | --- | --- | --- | --- | --- | --- |
| Buffer_100 | 1 | 55 | 54 | 0 | 0 | 0 | 0 | 0 | 0 | 1.3 | 0.0 | 1.8 | 0.7 | 0.1 | 0.0 | 0.0 | 9.4 | 3822.1 |
| Buffer_100 | 3 | 41 | 35 | 2 | 0 | 0 | 0 | 0 | 4 | 0.6 | 1.6 | 1.8 | 0.0 | 0.0 | 0.0 | 0.0 | 78.7 | 3807.6 |
| Buffer_100 | 3 | 20 | 15 | 0 | 0 | 0 | 3 | 0 | 2 | 0.4 | 2.0 | 0.6 | 0.0 | 0.9 | 302.0 | 1.0 | 65.5 | 4077.0 |
| Buffer_100 | 3 | 239 | 226 | 12 | 0 | 1 | 0 | 0 | 0 | 0.7 | 0.0 | 2.7 | 0.0 | 0.5 | 802.0 | 2.6 | 39.4 | 4364.6 |
| Buffer_100 | 2 | 151 | 147 | 0 | 0 | 0 | 0 | 0 | 4 | 0.6 | 1.0 | 1.4 | 0.0 | 0.9 | 431.0 | 1.4 | 50.9 | 2931.0 |
| Buffer_100 | 2 | 34 | 32 | 0 | 0 | 0 | 2 | 0 | 0 | 0.8 | 0.9 | 2.2 | 0.0 | 0.0 | 0.0 | 0.0 | 127.3 | 2101.2 |
| Buffer_100 | 4 | 14 | 7 | 2 | 0 | 1 | 4 | 0 | 0 | 0.4 | 0.5 | 1.9 | 0.0 | 1.1 | 277.0 | 0.9 | 194.1 | 2587.4 |
| Buffer_100 | 3 | 74 | 69 | 2 | 0 | 0 | 3 | 0 | 0 | 0.6 | 1.5 | 1.8 | 0.0 | 0.0 | 0.0 | 0.0 | 162.2 | 3574.1 |
| Buffer_100 | 2 | 6 | 1 | 5 | 0 | 0 | 0 | 0 | 0 | 0.1 | 0.2 | 2.5 | 0.0 | 1.1 | 0.0 | 0.0 | 132.3 | 4565.0 |
| Buffer_100 | 5 | 126 | 102 | 5 | 2 | 4 | 13 | 0 | 0 | 0.7 | 0.0 | 3.2 | 0.0 | 0.0 | 0.0 | 0.0 | 17.0 | 1684.5 |
| Buffer_100 | 6 | 349 | 235 | 42 | 48 | 14 | 8 | 0 | 2 | 0.2 | 0.1 | 0.5 | 0.0 | 3.2 | 0.0 | 0.0 | 67.5 | 154.5 |
| Buffer_100 | 4 | 19 | 15 | 1 | 0 | 0 | 2 | 0 | 1 | 0.5 | 0.7 | 1.8 | 0.0 | 0.9 | 1149.0 | 3.7 | 240.1 | 2144.1 |
| Buffer_100 | 1 | 1 | 1 | 0 | 0 | 0 | 0 | 0 | 0 | 0.0 | 1.5 | 2.4 | 0.0 | 0.0 | 372.0 | 1.2 | 291.2 | 2630.5 |
| Buffer_100 | 1 | 1 | 0 | 0 | 0 | 0 | 1 | 0 | 0 | 0.3 | 0.3 | 3.4 | 0.0 | 0.0 | 0.0 | 0.0 | 178.7 | 5774.4 |
| Buffer_100 | 0 | 0 | 0 | 0 | 0 | 0 | 0 | 0 | 0 | 0.2 | 0.2 | 2.3 | 0.3 | 1.0 | 0.0 | 0.0 | 111.3 | 6615.2 |
| Buffer_100 | 2 | 74 | 58 | 16 | 0 | 0 | 0 | 0 | 0 | 0.0 | 0.0 | 0.0 | 0.0 | 3.9 | 16007.0 | 51.6 | 63.6 | 1574.2 |
| Buffer_100 | 0 | 0 | 0 | 0 | 0 | 0 | 0 | 0 | 0 | 0.0 | 2.5 | 0.2 | 0.0 | 1.2 | 2950.0 | 9.5 | 373.1 | 2785.7 |
| Buffer_100 | 2 | 82 | 5 | 77 | 0 | 0 | 0 | 0 | 0 | 0.0 | 0.0 | 0.0 | 0.0 | 3.9 | 1055.0 | 3.4 | 615.7 | 3724.7 |
| Buffer_100 | 0 | 0 | 0 | 0 | 0 | 0 | 0 | 0 | 0 | 0.2 | 1.6 | 2.2 | 0.0 | 0.0 | 0.0 | 0.0 | 313.0 | 6055.3 |
| Buffer_100 | 2 | 3 | 0 | 0 | 0 | 2 | 1 | 0 | 0 | 0.8 | 1.3 | 1.8 | 0.0 | 0.0 | 0.0 | 0.0 | 204.3 | 7774.3 |
| Buffer_100 | 4 | 34 | 6 | 19 | 0 | 4 | 5 | 0 | 0 | 0.0 | 0.1 | 1.5 | 0.4 | 1.8 | 62.0 | 0.2 | 233.9 | 8529.0 |
| Buffer_100 | 4 | 34 | 9 | 13 | 6 | 6 | 0 | 0 | 0 | 0.4 | 0.2 | 1.7 | 0.0 | 1.5 | 824.0 | 2.6 | 81.8 | 2736.1 |
| Buffer_100 | 1 | 1 | 0 | 1 | 0 | 0 | 0 | 0 | 0 | 0.2 | 3.2 | 0.6 | 0.0 | 0.0 | 0.0 | 0.0 | 121.8 | 3066.1 |
| Buffer_100 | 2 | 3 | 0 | 0 | 1 | 2 | 0 | 0 | 0 | 0.3 | 2.6 | 0.5 | 0.0 | 0.6 | 1584.0 | 5.1 | 176.1 | 5721.3 |
| Buffer_100 | 2 | 14 | 0 | 8 | 0 | 0 | 6 | 0 | 0 | 0.2 | 2.4 | 1.0 | 0.0 | 0.3 | 1630.0 | 5.3 | 314.1 | 5702.0 |
| Buffer_100 | 0 | 0 | 0 | 0 | 0 | 0 | 0 | 0 | 0 | 0.0 | 0.0 | 0.0 | 0.0 | 3.9 | 186.0 | 0.6 | 271.7 | 8513.4 |
| Buffer_100 | 0 | 0 | 0 | 0 | 0 | 0 | 0 | 0 | 0 | 0.0 | 0.5 | 0.4 | 1.0 | 2.1 | 0.0 | 0.0 | 295.3 | 8589.7 |
| Buffer_100 | 1 | 2 | 2 | 0 | 0 | 0 | 0 | 0 | 0 | 0.6 | 0.0 | 1.8 | 0.0 | 1.5 | 876.0 | 2.9 | 80.0 | 4856.8 |
| Buffer_100 | 2 | 8 | 0 | 7 | 0 | 0 | 0 | 1 | 0 | 0.3 | 3.5 | 0.0 | 0.0 | 0.1 | 0.0 | 0.0 | 177.4 | 5698.6 |
| Buffer_100 | 0 | 0 | 0 | 0 | 0 | 0 | 0 | 0 | 0 | 0.0 | 0.2 | 0.7 | 0.0 | 3.0 | 429.0 | 1.4 | 240.2 | 7582.2 |
| Buffer_100 | 2 | 5 | 3 | 0 | 0 | 0 | 2 | 0 | 0 | 0.4 | 0.0 | 2.9 | 0.0 | 0.6 | 0.0 | 0.0 | 187.3 | 8326.4 |
| Buffer_300 | 1 | 55 | 54 | 0 | 0 | 0 | 0 | 0 | 0 | 11.0 | 0.0 | 8.8 | 3.0 | 12.5 | 110.0 | 0.0 | 9.4 | 3822.1 |
| Buffer_300 | 3 | 41 | 35 | 2 | 0 | 0 | 0 | 0 | 4 | 7.2 | 3.7 | 24.5 | 0.0 | 0.0 | 2026.0 | 0.7 | 78.3 | 3807.6 |
| Buffer_300 | 3 | 20 | 15 | 0 | 0 | 0 | 3 | 0 | 2 | 5.1 | 5.2 | 12.0 | 0.0 | 13.1 | 2507.0 | 0.9 | 71.5 | 4077.0 |
| Buffer_300 | 3 | 239 | 226 | 12 | 0 | 1 | 0 | 0 | 0 | 4.8 | 1.4 | 18.7 | 1.7 | 6.4 | 3164.0 | 1.1 | 42.9 | 4364.6 |
| Buffer_300 | 2 | 151 | 147 | 0 | 0 | 0 | 0 | 0 | 4 | 9.0 | 4.0 | 20.7 | 0.0 | 1.6 | 1406.0 | 0.5 | 52.8 | 2931.0 |
| Buffer_300 | 2 | 34 | 32 | 0 | 0 | 0 | 2 | 0 | 0 | 6.2 | 5.7 | 23.5 | 0.0 | 0.0 | 35.0 | 0.0 | 126.6 | 2101.2 |
| Buffer_300 | 4 | 14 | 7 | 2 | 0 | 1 | 4 | 0 | 0 | 5.0 | 2.1 | 8.8 | 0.0 | 19.5 | 7362.0 | 2.7 | 203.8 | 2587.4 |
| Buffer_300 | 3 | 74 | 69 | 2 | 0 | 0 | 3 | 0 | 0 | 3.7 | 17.8 | 13.9 | 0.0 | 0.0 | 0.0 | 0.0 | 161.6 | 3574.1 |
| Buffer_300 | 2 | 6 | 1 | 5 | 0 | 0 | 0 | 0 | 0 | 2.8 | 8.1 | 20.3 | 0.0 | 4.1 | 1652.0 | 0.6 | 130.8 | 4565.0 |
| Buffer_300 | 5 | 126 | 102 | 5 | 2 | 4 | 13 | 0 | 0 | 4.1 | 1.1 | 20.2 | 2.7 | 1.2 | 3336.0 | 1.2 | 16.5 | 1684.5 |
| Buffer_300 | 6 | 349 | 235 | 42 | 48 | 14 | 8 | 0 | 2 | 2.6 | 1.3 | 8.1 | 0.0 | 18.2 | 1188.0 | 0.4 | 76.0 | 154.5 |
| Buffer_300 | 4 | 19 | 15 | 1 | 0 | 0 | 2 | 0 | 1 | 2.3 | 8.1 | 13.5 | 0.0 | 11.4 | 29509.0 | 10.6 | 246.7 | 2144.1 |
| Buffer_300 | 1 | 1 | 1 | 0 | 0 | 0 | 0 | 0 | 0 | 1.2 | 7.8 | 20.7 | 0.0 | 5.7 | 16821.0 | 6.1 | 297.0 | 2630.5 |
| Buffer_300 | 1 | 1 | 0 | 0 | 0 | 0 | 1 | 0 | 0 | 7.4 | 3.3 | 24.6 | 0.0 | 0.0 | 427.0 | 0.2 | 191.8 | 5774.4 |
| Buffer_300 | 0 | 0 | 0 | 0 | 0 | 0 | 0 | 0 | 0 | 1.6 | 2.3 | 13.1 | 5.9 | 5.4 | 0.0 | 0.0 | 83.6 | 6615.2 |
| Buffer_300 | 2 | 74 | 58 | 16 | 0 | 0 | 0 | 0 | 0 | 0.6 | 0.0 | 0.3 | 1.5 | 32.9 | 80440.0 | 29.0 | 55.5 | 1574.2 |
| Buffer_300 | 0 | 0 | 0 | 0 | 0 | 0 | 0 | 0 | 0 | 0.0 | 17.1 | 2.7 | 0.0 | 15.5 | 40672.0 | 14.6 | 376.5 | 2785.7 |
| Buffer_300 | 2 | 82 | 5 | 77 | 0 | 0 | 0 | 0 | 0 | 0.0 | 0.0 | 0.0 | 1.5 | 33.8 | 13734.0 | 4.9 | 607.9 | 3724.7 |
| Buffer_300 | 0 | 0 | 0 | 0 | 0 | 0 | 0 | 0 | 0 | 1.2 | 8.0 | 20.9 | 0.0 | 5.2 | 2738.0 | 1.0 | 312.9 | 6055.3 |
| Buffer_300 | 2 | 3 | 0 | 0 | 0 | 2 | 1 | 0 | 0 | 3.8 | 7.4 | 24.2 | 0.0 | 0.0 | 0.0 | 0.0 | 203.4 | 7774.3 |
| Buffer_300 | 4 | 34 | 6 | 19 | 0 | 4 | 5 | 0 | 0 | 0.2 | 2.3 | 12.9 | 9.4 | 8.4 | 776.0 | 0.3 | 185.4 | 8529.0 |
| Buffer_300 | 4 | 34 | 9 | 13 | 6 | 6 | 0 | 0 | 0 | 4.2 | 3.9 | 9.5 | 0.8 | 16.9 | 18054.0 | 6.5 | 80.7 | 2736.1 |
| Buffer_300 | 1 | 1 | 0 | 1 | 0 | 0 | 0 | 0 | 0 | 5.6 | 16.5 | 13.3 | 0.0 | 0.0 | 861.0 | 0.3 | 124.9 | 3066.1 |
| Buffer_300 | 2 | 3 | 0 | 0 | 1 | 2 | 0 | 0 | 0 | 1.0 | 24.0 | 4.0 | 0.0 | 6.3 | 26557.0 | 9.6 | 180.2 | 5721.3 |
| Buffer_300 | 2 | 14 | 0 | 8 | 0 | 0 | 6 | 0 | 0 | 0.6 | 7.6 | 4.3 | 0.0 | 22.8 | 87173.0 | 31.4 | 325.5 | 5702.0 |
| Buffer_300 | 0 | 0 | 0 | 0 | 0 | 0 | 0 | 0 | 0 | 0.0 | 0.0 | 7.2 | 0.0 | 28.1 | 4930.0 | 1.8 | 260.6 | 8513.4 |
| Buffer_300 | 0 | 0 | 0 | 0 | 0 | 0 | 0 | 0 | 0 | 0.0 | 1.9 | 6.4 | 10.8 | 16.1 | 746.0 | 0.3 | 272.1 | 8589.7 |
| Buffer_300 | 1 | 2 | 2 | 0 | 0 | 0 | 0 | 0 | 0 | 2.8 | 8.4 | 12.3 | 0.5 | 11.1 | 8482.0 | 3.1 | 66.1 | 4856.8 |
| Buffer_300 | 2 | 8 | 0 | 7 | 0 | 0 | 0 | 1 | 0 | 1.1 | 27.2 | 4.1 | 0.0 | 2.9 | 3711.0 | 1.3 | 184.0 | 5698.6 |
| Buffer_300 | 0 | 0 | 0 | 0 | 0 | 0 | 0 | 0 | 0 | 0.1 | 4.9 | 10.5 | 0.0 | 19.8 | 4904.0 | 1.8 | 241.7 | 7582.2 |
| Buffer_300 | 2 | 5 | 3 | 0 | 0 | 0 | 2 | 0 | 0 | 1.3 | 1.0 | 25.8 | 0.0 | 7.2 | 1704.0 | 0.6 | 174.3 | 8326.4 |

Table S2 – GLMM Results from single-variable models of Total Bat Species for 100m and 300m Buffers. Bold *p-value*: significant values (*<0.05).

| **Total Species** | **Buffer 100** | | | | | **Buffer 300** | | | | |
| --- | --- | --- | --- | --- | --- | --- | --- | --- | --- | --- |
|  | **Estimate** | **Std. Error** | **z value** | ***p-value*** | **AIC** | **Estimate** | **Std. Error** | **z value** | ***p-value*** | **AIC** |
| **(Intercept)** | 0.674 | 0.154 | 4.38 | **1.2e-05** | 114.6 | 0.678 | 0.154 | 4.39 | **1.2e-05** | 116.0 |
| **Urban** | 0.211 | 0.132 | 1.61 | 0.110 |  | 0.148 | 0.134 | 1.1 | 0.270 |  |
| **(Intercept)** | 0.685 | 0.151 | 4.53 | **5.8e-06** | 116.3 | 0.685 | 0.152 | 4.52 | **6.3e-06** | 116.5 |
| **Vineyard** | -0.135 | 0.145 | -0.94 | 0.350 |  | -0.118 | 0.147 | -0.8 | 0.420 |  |
| **(Intercept)** | 0.681 | 0.153 | 4.44 | **9.0e-06** | 116.2 | 0.682 | 0.154 | 4.42 | **9.9e-06** | 117.0 |
| **Complex_cultivation_patterns** | 0.134 | 0.137 | 0.97 | 0.330 |  | 0.050 | 0.139 | 0.36 | 0.720 |  |
| **(Intercept)** | 0.671 | 0.154 | 4.36 | **1.3e-05** | 114.8 | 0.681 | 0.155 | 4.41 | **1.0e-05** | 117.0 |
| **Bare_rocks** | -0.264 | 0.192 | -1.38 | 0.170 |  | -0.062 | 0.149 | -0.42 | 0.680 |  |
| **(Intercept)** | 0.682 | 0.154 | 4.41 | **1.0e-05** | 117.2 | 0.682 | 0.154 | 4.42 | **9.9e-06** | 117.1 |
| **Mediterranean_vegetation** | -0.024 | 0.141 | -0.17 | 0.870 |  | -0.039 | 0.141 | -0.28 | 0.780 |  |
| **(Intercept)** | 0.683 | 0.154 | 4.44 | **9.0e-06** | 117.1 | 0.683 | 0.154 | 4.44 | **9.1e-06** | 117.2 |
| **TCD_sum** | -0.026 | 0.145 | -0.18 | 0.860 |  | -0.017 | 0.141 | -0.12 | 0.900 |  |
| **(Intercept)** | 0.683 | 0.154 | 4.44 | **9.0e-06** | 117.1 | 0.683 | 0.154 | 4.44 | **9.1e-06** | 117.2 |
| **TCD_mean** | -0.026 | 0.145 | -0.18 | 0.860 |  | -0.017 | 0.141 | -0.12 | 0.900 |  |
| **(Intercept)** | 0.682 | 0.131 | 5.2 | **2.0e-07** | 112.7 | 0.685 | 0.144 | 4.74 | **2.1e-06** | 113.0 |
| **Altitude** | -0.319 | 0.153 | -2.09 | **0.037** |  | -0.305 | 0.152 | -2.01 | **0.045** |  |
| **(Intercept)** | 0.642 | 0.136 | 4.73 | **2.2e-06** | 107.6 | 0.642 | 0.136 | 4.73 | **2.2e-06** | 107.6 |
| **d_water** | -0.424 | 0.140 | -3.04 | **0.002** |  | -0.424 | 0.140 | -3.04 | **0.002** |  |

Table S3 – GLMM Results from single-model Models of Total Bat Passes for 100m and 300m Buffers. Bold *p-value*: significant values (*<0.05).

| **Total Passes** | **Buffer 100** | | | | | **Buffer 300** | | | | |
| --- | --- | --- | --- | --- | --- | --- | --- | --- | --- | --- |
|  | **Estimate** | **Std. Error** | **z value** | ***p-value*** | **AIC** | **Estimate** | **Std. Error** | **z value** | ***p-value*** | **AIC** |
| **(Intercept)** | 2.049 | 0.406 | 5.05 | **4.5e-07** | 273.8 | 2.060 | 0.414 | 4.97 | **6.7e-07** | 275.9 |
| **Urban** | 1.129 | 0.405 | 2.79 | **0.005** |  | 0.981 | 0.412 | 2.38 | **0.017** |  |
| **(Intercept)** | 2.096 | 0.424 | 4.95 | **7.6e-07** | 279.7 | 2.101 | 0.420 | 5.00 | **5.8e-07** | 279.5 |
| **Vineyard** | -0.569 | 0.423 | -1.34 | 0.180 |  | -0.594 | 0.415 | -1.43 | 0.150 |  |
| **(Intercept)** | 2.070 | 0.443 | 4.67 | **3.0e-06** | 281.1 | 2.076 | 0.443 | 4.69 | **2.7e-06** | 281.5 |
| **Complex_cultivation_patterns** | 0.254 | 0.445 | 0.57 | 0.570 |  | 0.021 | 0.437 | 0.05 | 0.960 |  |
| **(Intercept)** | 2.061 | 0.443 | 4.65 | **3.4e-06** | 280.7 | 2.067 | 0.445 | 4.65 | **3.3e-06** | 281.2 |
| **Bare_rocks** | -0.409 | 0.484 | -0.84 | 0.400 |  | -0.224 | 0.476 | -0.47 | 0.640 |  |
| **(Intercept)** | 2.078 | 0.442 | 4.70 | **2.6e-06** | 281.5 | 2.079 | 0.4413 | 4.71 | **2.5e-06** | 281.4 |
| **Mediterranean_vegetation** | 0.039 | 0.445 | 0.09 | 0.930 |  | 0.076 | 0.4353 | 0.18 | 0.860 |  |
| **(Intercept)** | 2.079 | 0.437 | 4.76 | **1.9e-06** | 280.8 | 2.075 | 0.442 | 4.69 | **2.7e-06** | 281.4 |
| **TCD_sum** | 0.353 | 0.416 | 0.85 | 0.400 |  | 0.145 | 0.427 | 0.34 | 0.730 |  |
| **(Intercept)** | 2.079 | 0.437 | 4.76 | **1.9e-06** | 280.8 | 2.075 | 0.442 | 4.69 | **2.7e-06** | 281.4 |
| **TCD_mean** | 0.352 | 0.416 | 0.85 | 0.400 |  | 0.146 | 0.427 | 0.34 | 0.730 |  |
| **(Intercept)** | 2.067 | 0.416 | 4.96 | **6.9e-07** | 276.6 | 2.069 | 0.417 | 4.96 | **7.1e-07** | 277.0 |
| **Altitude** | -0.912 | 0.412 | -2.22 | **0.027** |  | -0.885 | 0.413 | -2.14 | **0.032** |  |
| **(Intercept)** | 2.088 | 0.361 | 5.78 | **7.3e-09** | 268.3 | 2.088 | 0.361 | 5.78 | **7.3e-09** | 268.3 |
| **d_water** | -1.426 | 0.371 | -3.85 | **1.2e-04** |  | -1.426 | 0.371 | -3.85 | **1.2e-04** |  |

Table S4 – GLMM Results from single-variable models of *Pipistrellus kuhlii* for 100m and 300m Buffers. Bold *p-value*: significant values (*<0.05).

| ***P. kuhlii*** | **Buffer 100** | | | | | **Buffer 300** | | | | |
| --- | --- | --- | --- | --- | --- | --- | --- | --- | --- | --- |
|  | **Estimate** | **Std. Error** | **z value** | ***p-value*** | **AIC** | **Estimate** | **Std. Error** | **z value** | ***p-value*** | **AIC** |
| **(Intercept)** | 0.826 | 0.573 | 1.44 | 0.149 | 226.6 | 0.822 | 0.583 | 1.41 | 0.159 | 227.8 |
| **Urban** | 1.633 | 0.521 | 3.14 | **0.002** |  | 1.560 | 0.534 | 2.92 | **0.004** |  |
| **(Intercept)** | 0.636 | 0.642 | 0.99 | 0.321 | 229.1 | 0.686 | 0.624 | 1.1 | 0.272 | 229.2 |
| **Vineyard** | -1.725 | 0.679 | -2.54 | **0.011** |  | -1.717 | 0.694 | -2.47 | **0.013** |  |
| **(Intercept)** | 0.713 | 0.677 | 1.05 | 0.290 | 234.4 | 0.753 | 0.667 | 1.13 | 0.260 | 235.4 |
| **Complex_cultivation_patterns** | 0.840 | 0.644 | 1.3 | 0.190 |  | 0.547 | 0.621 | 0.88 | 0.380 |  |
| **(Intercept)** | 0.766 | 0.666 | 1.15 | 0.250 | 236.0 | 0.771 | 0.664 | 1.16 | 0.250 | 236.1 |
| **Bare_rocks** | -0.284 | 0.657 | -0.43 | 0.670 |  | -0.163 | 0.652 | -0.25 | 0.800 |  |
| **(Intercept)** | 0.770 | 0.665 | 1.16 | 0.250 | 236.1 | 0.7731 | 0.6642 | 1.16 | 0.240 | 236.2 |
| **Mediterranean_vegetation** | 0.154 | 0.612 | 0.25 | 0.800 |  | -0.0243 | 0.6071 | -0.04 | 0.970 |  |
| **(Intercept)** | 0.780 | 0.656 | 1.19 | 0.230 | 235.5 | 0.766 | 0.665 | 1.15 | 0.250 | 235.9 |
| **TCD_sum** | 0.479 | 0.554 | 0.86 | 0.390 |  | -0.332 | 0.646 | -0.51 | 0.610 |  |
| **(Intercept)** | 0.780 | 0.656 | 1.19 | 0.230 | 235.5 | 0.766 | 0.665 | 1.15 | 0.250 | 235.9 |
| **TCD_mean** | 0.478 | 0.554 | 0.86 | 0.390 |  | -0.332 | 0.647 | -0.51 | 0.610 |  |
| **(Intercept)** | 0.881 | 0.560 | 1.57 | 0.116 | 227.1 | 0.869 | 0.565 | 1.54 | 0.124 | 227.2 |
| **Altitude** | -1.575 | 0.505 | -3.12 | **0.002** |  | -1.575 | 0.511 | -3.08 | **0.002** |  |
| **(Intercept)** | 0.825 | 0.522 | 1.58 | 0.110 | 219.6 | 0.825 | 0.522 | 1.58 | 0.110 | 219.6 |
| **d_water** | -2.092 | 0.509 | -4.11 | **3.9e-05** |  | -2.092 | 0.509 | -4.11 | **3.9e-05** |  |

Table S5 – GLMM Results from single-variable models of *Hypsugo savii* for 100m and 300m Buffers. Bold *p-value*: significant values (*<0.05).

| ***H. savii*** | **Buffer 100** | | | | | **Buffer 300** | | | | |
| --- | --- | --- | --- | --- | --- | --- | --- | --- | --- | --- |
|  | **Estimate** | **Std. Error** | **z value** | ***p-value*** | **AIC** | **Estimate** | **Std. Error** | **z value** | ***p-value*** | **AIC** |
| **(Intercept)** | -0.458 | 0.673 | -0.68 | 0.500 | 158.0 | -0.443 | 0.665 | -0.67 | 0.510 | 157.9 |
| **Urban** | -0.597 | 0.574 | -1.04 | 0.300 |  | -0.633 | 0.579 | -1.09 | 0.270 |  |
| **(Intercept)** | -0.450 | 0.677 | -0.66 | 0.510 | 158.4 | -0.462 | 0.684 | -0.68 | 0.500 | 158.6 |
| **Vineyard** | -0.431 | 0.525 | -0.82 | 0.410 |  | -0.352 | 0.532 | -0.66 | 0.510 |  |
| **(Intercept)** | -0.460 | 0.680 | -0.68 | 0.500 | 158.2 | -0.400 | 0.626 | -0.64 | 0.523 | 154.9 |
| **Complex_cultivation_patterns** | -0.509 | 0.541 | -0.94 | 0.350 |  | -1.066 | 0.516 | -2.06 | **0.039** |  |
| **(Intercept)** | -0.544 | 0.700 | -0.78 | 0.440 | 157.9 | -0.487 | 0.694 | -0.70 | 0.480 | 158.8 |
| **Bare_rocks** | -0.692 | 0.661 | -1.05 | 0.300 |  | 0.265 | 0.559 | 0.47 | 0.630 |  |
| **(Intercept)** | -0.318 | 0.611 | -0.52 | 0.600 | 155.6 | -0.342 | 0.617 | -0.55 | 0.580 | 155.5 |
| **Mediterranean_vegetation** | 0.916 | 0.466 | 1.96 | **0.050** |  | 0.926 | 0.468 | 1.98 | **0.048** |  |
| **(Intercept)** | -0.475 | 0.680 | -0.70 | 0.490 | 157.4 | -0.498 | 0.687 | -0.73 | 0.470 | 157.1 |
| **TCD_sum** | 0.605 | 0.475 | 1.27 | 0.200 |  | 0.688 | 0.498 | 1.38 | 0.170 |  |
| **(Intercept)** | -0.475 | 0.680 | -0.70 | 0.490 | 157.4 | -0.498 | 0.687 | -0.73 | 0.470 | 157.1 |
| **TCD_mean** | 0.605 | 0.475 | 1.27 | 0.200 |  | 0.688 | 0.498 | 1.38 | 0.170 |  |
| **(Intercept)** | -0.494 | 0.701 | -0.70 | 0.480 | 159.0 | -0.496 | 0.701 | -0.71 | 0.480 | 159.0 |
| **Altitude** | 0.117 | 0.526 | 0.22 | 0.820 |  | 0.110 | 0.527 | 0.21 | 0.840 |  |
| **(Intercept)** | -0.585 | 0.692 | -0.85 | 0.398 | 154.5 | -0.585 | 0.692 | -0.85 | 0.398 | 154.5 |
| **d_water** | -1.184 | 0.590 | -2.01 | **0.045** |  | -1.184 | 0.590 | -2.01 | **0.045** |  |

Table S6 – GLMM Results from single-variable Models of Total Bat Species, with each explanatory variable interacting with the “distance to water”, for 100m and 300m Buffers. Bold *p-value*: significant values (*<0.05).

| **Total Species** | **Buffer 100** | | | | | **Buffer 300** | | | | |
| --- | --- | --- | --- | --- | --- | --- | --- | --- | --- | --- |
|  | **Estimate** | **Std. Error** | **z value** | ***p-value*** | **AIC** | **Estimate** | **Std. Error** | **z value** | ***p-value*** | **AIC** |
| **(Intercept)** | 0.631 | 0.141 | 4.46 | **8.0e-06** | 110.5 | 0.595 | 0.162 | 3.66 | **2.5e-04** | 111.3 |
| **Urban** | 0.135 | 0.138 | 0.98 | 0.327 |  | -0.017 | 0.156 | -0.11 | 0.912 |  |
| **d_water** | -0.404 | 0.149 | -2.71 | **0.007** |  | -0.472 | 0.175 | -2.70 | **0.007** |  |
| **Urban:d_water** | 0.005 | 0.168 | 0.03 | 0.978 |  | -0.128 | 0.231 | -0.55 | 0.580 |  |
| **(Intercept)** | 0.633 | 0.136 | 4.65 | **3.3e-06** | 108.2 | 0.630 | 0.137 | 4.61 | **4.0e-06** | 109.2 |
| **Vineyard** | -0.094 | 0.139 | -0.67 | 0.500 |  | -0.091 | 0.137 | -0.66 | 0.508 |  |
| **d_water** | -0.262 | 0.165 | -1.59 | 0.110 |  | -0.282 | 0.167 | -1.69 | 0.091 |  |
| **Vineyard:d_water** | 0.324 | 0.200 | 1.62 | 0.110 |  | 0.290 | 0.205 | 1.42 | 0.157 |  |
| **(Intercept)** | 0.628 | 0.138 | 4.55 | **5.5e-06** | 110.3 | 0.624 | 0.139 | 4.49 | **7.1e-06** | 110.6 |
| **Complex_cultivation_patterns** | 0.159 | 0.138 | 1.15 | 0.251 |  | 0.119 | 0.136 | 0.87 | 0.382 |  |
| **d_water** | -0.428 | 0.142 | -3.01 | **0.003** |  | -0.435 | 0.141 | -3.09 | **0.002** |  |
| **Complex_cultivation_patterns:d_water** | 0.064 | 0.131 | 0.49 | 0.624 |  | 0.107 | 0.137 | 0.78 | 0.435 |  |
| **(Intercept)** | 0.571 | 0.162 | 3.53 | **4.1e-04** | 110.3 | 0.604 | 0.154 | 3.91 | **9.1e-05** | 110.7 |
| **Bare_rocks** | -0.262 | 0.290 | -0.90 | 0.366 |  | 0.037 | 0.260 | 0.14 | 0.887 |  |
| **d_water** | -0.366 | 0.154 | -2.38 | **0.017** |  | -0.463 | 0.152 | -3.04 | **0.002** |  |
| **Bare_rocks:d_water** | 0.179 | 0.202 | 0.89 | 0.375 |  | 0.085 | 0.171 | 0.49 | 0.621 |  |
| **(Intercept)** | 0.629 | 0.139 | 4.52 | **6.0e-06** | 110.1 | 0.608 | 0.142 | 4.27 | **2.0e-05** | 109.5 |
| **Mediterranean_vegetation** | -0.138 | 0.161 | -0.85 | 0.394 |  | -0.188 | 0.164 | -1.15 | 0.251 |  |
| **d_water** | -0.370 | 0.152 | -2.43 | **0.015** |  | -0.409 | 0.145 | -2.83 | **0.005** |  |
| **Mediterranean_vegetation:d_water** | -0.160 | 0.137 | -1.17 | 0.243 |  | -0.215 | 0.161 | -1.33 | 0.183 |  |
| **(Intercept)** | 0.598 | 0.202 | 2.97 | **0.003** | 110.6 | 0.658 | 0.137 | 4.80 | **1.6e-06** | 110.5 |
| **TCD_sum** | -0.304 | 0.713 | -0.43 | 0.670 |  | -0.035 | 0.147 | -0.24 | 0.811 |  |
| **d_water** | -0.491 | 0.203 | -2.41 | **0.016** |  | -0.418 | 0.143 | -2.93 | **0.003** |  |
| **TCD_sum:d_water** | -0.145 | 0.566 | -0.26 | 0.798 |  | 0.121 | 0.157 | 0.77 | 0.444 |  |
| **(Intercept)** | 0.598 | 0.201 | 2.97 | **0.003** | 110.6 | 0.658 | 0.137 | 4.80 | **1.6e-06** | 110.5 |
| **TCD_mean** | -0.303 | 0.710 | -0.43 | 0.669 |  | -0.035 | 0.147 | -0.24 | 0.812 |  |
| **d_water** | -0.491 | 0.203 | -2.42 | **0.016** |  | -0.418 | 0.143 | -2.93 | **0.003** |  |
| **TCD_mean:d_water** | -0.144 | 0.564 | -0.26 | 0.798 |  | 0.121 | 0.157 | 0.77 | 0.444 |  |
| **(Intercept)** | 0.600 | 0.148 | 4.06 | **5.0e-05** | 109.9 | 0.623 | 0.144 | 4.34 | **1.4e-05** | 110.1 |
| **Altitude** | -0.114 | 0.175 | -0.65 | 0.514 |  | -0.154 | 0.174 | -0.89 | 0.376 |  |
| **d_water** | -0.364 | 0.145 | -2.51 | **0.012** |  | -0.372 | 0.146 | -2.55 | **0.011** |  |
| **Altitude:d_water** | 0.120 | 0.206 | 0.58 | 0.562 |  | 0.037 | 0.218 | 0.17 | 0.864 |  |

Table S7 – GLMM Results from single-variable Models of Total Bat Passes, with each explanatory variable interacting with the “distance to water”, for 100m and 300m Buffers. Bold *p-value*: significant values (*<0.05).

| **Total Passes** | **Buffer 100** | | | | | **Buffer 300** | | | | |
| --- | --- | --- | --- | --- | --- | --- | --- | --- | --- | --- |
|  | **Estimate** | **Std. Error** | **z value** | ***p-value*** | **AIC** | **Estimate** | **Std. Error** | **z value** | ***p-value*** | **AIC** |
| **(Intercept)** | 2.053 | 0.348 | 5.91 | **3.5e-09** | 266.9 | 1.936 | 0.395 | 4.90 | **9.8e-07** | 269.7 |
| **Urban** | 0.774 | 0.351 | 2.21 | **0.027** |  | 0.379 | 0.389 | 0.97 | 0.330 |  |
| **d_water** | -1.256 | 0.363 | -3.46 | **0.001** |  | -1.432 | 0.445 | -3.22 | **0.001** |  |
| **Urban:d_water** | -0.078 | 0.396 | -0.20 | 0.844 |  | -0.455 | 0.566 | -0.80 | 0.422 |  |
| **(Intercept)** | 2.151 | 0.322 | 6.68 | **2.4e-11** | 265.6 | 2.125 | 0.323 | 6.58 | **4.8e-11** | 265.7 |
| **Vineyard** | -0.582 | 0.319 | -1.82 | 0.068 |  | -0.659 | 0.321 | -2.06 | **0.040** |  |
| **d_water** | -0.949 | 0.380 | -2.50 | **0.012** |  | -0.926 | 0.383 | -2.42 | **0.016** |  |
| **Vineyard:d_water** | 0.988 | 0.475 | 2.08 | **0.038** |  | 1.023 | 0.501 | 2.04 | **0.041** |  |
| **(Intercept)** | 2.073 | 0.361 | 5.74 | **9.4e-09** | 271.2 | 2.055 | 0.366 | 5.62 | **1.9e-08** | 271.7 |
| **Complex_cultivation_patterns** | 0.256 | 0.361 | 0.71 | 0.478 |  | 0.149 | 0.352 | 0.42 | 0.673 |  |
| **d_water** | -1.417 | 0.372 | -3.80 | **1.4e-04** |  | -1.456 | 0.377 | -3.86 | **1.1e-04** |  |
| **Complex_cultivation_patterns:d_water** | 0.290 | 0.358 | 0.81 | 0.418 |  | 0.248 | 0.365 | 0.68 | 0.497 |  |
| **(Intercept)** | 2.148 | 0.376 | 5.71 | **1.1e-08** | 271.9 | 2.277 | 0.381 | 5.97 | **2.3e-09** | 270.1 |
| **Bare_rocks** | 0.283 | 0.488 | 0.58 | 0.562 |  | 1.014 | 0.720 | 1.41 | 0.160 |  |
| **d_water** | -1.475 | 0.386 | -3.82 | **1.3e-04** |  | -1.614 | 0.377 | -4.28 | **1.8e-05** |  |
| **Bare_rocks:d_water** | -0.169 | 0.397 | -0.43 | 0.670 |  | -0.409 | 0.476 | -0.86 | 0.390 |  |
| **(Intercept)** | 2.078 | 0.361 | 5.76 | **8.5e-09** | 270.2 | 2.042 | 0.367 | 5.57 | **2.5e-08** | 270.9 |
| **Mediterranean_vegetation** | -0.105 | 0.389 | -0.27 | 0.788 |  | -0.163 | 0.385 | -0.42 | 0.673 |  |
| **d_water** | -1.239 | 0.387 | -3.20 | **0.001** |  | -1.370 | 0.378 | -3.63 | **2.9e-04** |  |
| **Mediterranean_vegetation:d_water** | -0.495 | 0.357 | -1.39 | 0.165 |  | -0.468 | 0.408 | -1.15 | 0.252 |  |
| **(Intercept)** | 2.009 | 0.491 | 4.09 | **4.2e-05** | 272.2 | 2.133 | 0.364 | 5.87 | **4.5e-09** | 271.8 |
| **TCD_sum** | -0.397 | 1.668 | -0.24 | 0.812 |  | -0.031 | 0.361 | -0.09 | 0.931 |  |
| **d_water** | -1.512 | 0.520 | -2.91 | **0.004** |  | -1.411 | 0.376 | -3.75 | **1.8e-04** |  |
| **TCD_sum:d_water** | -0.313 | 1.312 | -0.24 | 0.812 |  | 0.244 | 0.386 | 0.63 | 0.527 |  |
| **(Intercept)** | 2.009 | 0.490 | 4.10 | **4.1e-05** | 272.2 | 2.133 | 0.364 | 5.87 | **4.5e-09** | 271.8 |
| **TCD_mean** | -0.400 | 1.660 | -0.24 | 0.810 |  | -0.031 | 0.361 | -0.09 | 0.932 |  |
| **d_water** | -1.512 | 0.518 | -2.92 | **0.004** |  | -1.411 | 0.376 | -3.75 | **1.8e-04** |  |
| **TCD_mean:d_water** | -0.315 | 1.306 | -0.24 | 0.810 |  | 0.244 | 0.386 | 0.63 | 0.527 |  |
| **(Intercept)** | 1.983 | 0.374 | 5.31 | **1.1e-07** | 269.7 | 2.040 | 0.367 | 5.56 | **2.6e-08** | 269.8 |
| **Altitude** | -0.311 | 0.415 | -0.75 | 0.453 |  | -0.420 | 0.417 | -1.01 | 0.314 |  |
| **d_water** | -1.302 | 0.368 | -3.54 | **4.0e-04** |  | -1.287 | 0.364 | -3.54 | **4.0e-04** |  |
| **Altitude:d_water** | 0.451 | 0.579 | 0.78 | 0.436 |  | 0.261 | 0.603 | 0.43 | 0.666 |  |

Table S8 – GLMM Results from single-variable models of *Pipistrellus kuhlii*, with each explanatory variable interacting with the “distance to water”, for 100m and 300m Buffers. Bold *p-value*: significant values (*<0.05).

| ***P. kuhlii*** | **Buffer 100** | | | | | **Buffer 300** | | | | |
| --- | --- | --- | --- | --- | --- | --- | --- | --- | --- | --- |
|  | **Estimate** | **Std. Error** | **z value** | ***p-value*** | **AIC** | **Estimate** | **Std. Error** | **z value** | ***p-value*** | **AIC** |
| **(Intercept)** | 0.826 | 0.463 | 1.79 | 0.074 | 215.2 | 0.533 | 0.557 | 0.96 | 0.339 | 217.5 |
| **Urban** | 0.988 | 0.416 | 2.38 | **0.017** |  | 0.555 | 0.481 | 1.15 | 0.248 |  |
| **d_water** | -1.844 | 0.458 | -4.02 | **5.8e-05** |  | -2.188 | 0.598 | -3.66 | **2.5e-04** |  |
| **Urban:d_water** | -0.468 | 0.499 | -0.94 | 0.349 |  | -1.048 | 0.735 | -1.43 | 0.154 |  |
| **(Intercept)** | 0.666 | 0.540 | 1.23 | 0.217 | 213.7 | 0.629 | 0.565 | 1.11 | 0.266 | 214 |
| **Vineyard** | -1.608 | 0.603 | -2.67 | **0.008** |  | -1.830 | 0.787 | -2.33 | **0.020** |  |
| **d_water** | -2.350 | 0.697 | -3.37 | **0.001** |  | -2.401 | 0.774 | -3.10 | **0.002** |  |
| **Vineyard:d_water** | -0.604 | 0.881 | -0.69 | 0.493 |  | -0.826 | 1.119 | -0.74 | 0.460 |  |
| **(Intercept)** | 0.722 | 0.530 | 1.36 | 0.173 | 219.8 | 0.749 | 0.523 | 1.43 | 0.152 | 220.5 |
| **Complex_cultivation_patterns** | 0.908 | 0.508 | 1.79 | 0.074 |  | 0.790 | 0.474 | 1.67 | 0.096 |  |
| **d_water** | -2.181 | 0.527 | -4.14 | **3.5e-05** |  | -2.217 | 0.520 | -4.26 | **2.0e-05** |  |
| **Complex_cultivation_patterns:d_water** | 0.507 | 0.495 | 1.02 | 0.306 |  | 0.395 | 0.482 | 0.82 | 0.413 |  |
| **(Intercept)** | 0.888 | 0.530 | 1.67 | 0.094 | 222.1 | 1.025 | 0.533 | 1.92 | 0.054 | 219.9 |
| **Bare_rocks** | 0.674 | 0.594 | 1.13 | 0.257 |  | 1.465 | 0.896 | 1.64 | 0.102 |  |
| **d_water** | -2.262 | 0.527 | -4.30 | **1.7e-05** |  | -2.438 | 0.532 | -4.58 | **4.7e-06** |  |
| **Bare_rocks:d_water** | -0.110 | 0.484 | -0.23 | 0.821 |  | -0.431 | 0.588 | -0.73 | 0.464 |  |
| **(Intercept)** | 0.827 | 0.523 | 1.58 | 0.114 | 223.5 | 0.812 | 0.521 | 1.56 | 0.120 | 223.1 |
| **Mediterranean_vegetation** | 0.064 | 0.504 | 0.13 | 0.900 |  | -0.349 | 0.546 | -0.64 | 0.520 |  |
| **d_water** | -2.074 | 0.549 | -3.78 | **1.6e-04** |  | -2.077 | 0.513 | -4.05 | **5.2e-05** |  |
| **Mediterranean_vegetation:d_water** | -0.034 | 0.463 | -0.07 | 0.942 |  | -0.263 | 0.580 | -0.45 | 0.650 |  |
| **(Intercept)** | -0.117 | 0.832 | -0.14 | 0.888 | 220.5 | -0.906 | 1.016 | -0.89 | 0.373 | 214.3 |
| **TCD_sum** | -4.555 | 2.802 | -1.63 | 0.104 |  | -5.519 | 2.236 | -2.47 | **0.014** |  |
| **d_water** | -3.081 | 0.829 | -3.72 | **2.0e-04** |  | -3.998 | 1.004 | -3.98 | **6.8e-05** |  |
| **TCD_sum:d_water** | -3.586 | 2.198 | -1.63 | 0.103 |  | -4.225 | 1.825 | -2.32 | **0.021** |  |
| **(Intercept)** | -0.116 | 0.831 | -0.14 | 0.889 | 220.5 | -0.906 | 1.017 | -0.89 | 0.373 | 214.3 |
| **TCD_mean** | -4.553 | 2.793 | -1.63 | 0.103 |  | -5.520 | 2.237 | -2.47 | **0.014** |  |
| **d_water** | -3.080 | 0.827 | -3.73 | **2.0e-04** |  | -3.997 | 1.004 | -3.98 | **6.8e-05** |  |
| **TCD_mean:d_water** | -3.583 | 2.191 | -1.64 | 0.102 |  | -4.226 | 1.826 | -2.31 | **0.021** |  |
| **(Intercept)** | 0.888 | 0.473 | 1.88 | 0.060 | 217.5 | 0.965 | 0.456 | 2.11 | **0.034** | 216.7 |
| **Altitude** | -0.880 | 0.487 | -1.81 | 0.071 |  | -1.120 | 0.502 | -2.23 | **0.026** |  |
| **d_water** | -1.685 | 0.446 | -3.78 | **1.6e-04** |  | -1.717 | 0.444 | -3.86 | **1.1e-04** |  |
| **Altitude:d_water** | 0.316 | 0.683 | 0.46 | 0.644 |  | -0.112 | 0.732 | -0.15 | 0.878 |  |

Table S9 – GLMM Results from single-variable models of *Hypsugo savii*, with each explanatory variable interacting with the “distance to water”, for 100m and 300m Buffers. Bold *p-value*: significant values (*<0.05).

| ***H. savii*** | **Buffer 100** | | | | | **Buffer 300** | | | | |
| --- | --- | --- | --- | --- | --- | --- | --- | --- | --- | --- |
|  | **Estimate** | **Std. Error** | **z value** | ***p-value*** | **AIC** | **Estimate** | **Std. Error** | **z value** | ***p-value*** | **AIC** |
| **(Intercept)** | -0.567 | 0.691 | -0.82 | 0.412 | 155.7 | -0.573 | 0.722 | -0.79 | 0.427 | 154.2 |
| **Urban** | -0.991 | 0.616 | -1.61 | 0.108 |  | -1.281 | 0.656 | -1.95 | 0.051 |  |
| **d_water** | -1.431 | 0.686 | -2.09 | **0.037** |  | -1.626 | 0.765 | -2.13 | **0.034** |  |
| **Urban:d_water** | -0.216 | 0.722 | -0.30 | 0.765 |  | -0.318 | 0.928 | -0.34 | 0.732 |  |
| **(Intercept)** | -0.344 | 0.589 | -0.58 | 0.560 | 154 | -0.407 | 0.612 | -0.66 | 0.506 | 155.4 |
| **Vineyard** | -0.434 | 0.462 | -0.94 | 0.350 |  | -0.409 | 0.478 | -0.86 | 0.392 |  |
| **d_water** | -0.376 | 0.593 | -0.63 | 0.530 |  | -0.498 | 0.617 | -0.81 | 0.420 |  |
| **Vineyard:d_water** | 1.437 | 0.700 | 2.05 | **0.040** |  | 1.284 | 0.746 | 1.72 | 0.085 |  |
| **(Intercept)** | -0.494 | 0.664 | -0.74 | 0.457 | 157.3 | -0.447 | 0.618 | -0.72 | 0.469 | 154.6 |
| **Complex_cultivation_patterns** | -0.475 | 0.525 | -0.90 | 0.366 |  | -0.945 | 0.503 | -1.88 | 0.061 |  |
| **d_water** | -1.096 | 0.578 | -1.90 | 0.058 |  | -0.970 | 0.536 | -1.81 | 0.071 |  |
| **Complex_cultivation_patterns:d_water** | 0.231 | 0.528 | 0.44 | 0.661 |  | 0.215 | 0.553 | 0.39 | 0.697 |  |
| **(Intercept)** | -1.966 | 2.547 | -0.77 | 0.440 | 155.7 | -0.4751 | 0.6980 | -0.68 | 0.496 | 155.9 |
| **Bare_rocks** | -4.331 | 7.188 | -0.60 | 0.550 |  | 1.0652 | 1.0794 | 0.99 | 0.324 |  |
| **d_water** | -0.288 | 1.639 | -0.18 | 0.860 |  | -1.5429 | 0.6281 | -2.46 | **0.014** |  |
| **Bare_rocks:d_water** | 2.897 | 4.273 | 0.68 | 0.500 |  | -0.0938 | 0.7072 | -0.13 | 0.894 |  |
| **(Intercept)** | -0.243 | 0.573 | -0.42 | 0.670 | 153.5 | -0.350 | 0.593 | -0.59 | 0.550 | 153.9 |
| **Mediterranean_vegetation** | 0.691 | 0.453 | 1.52 | 0.130 |  | 0.622 | 0.473 | 1.31 | 0.190 |  |
| **d_water** | -0.629 | 0.537 | -1.17 | 0.240 |  | -0.776 | 0.520 | -1.49 | 0.140 |  |
| **Mediterranean_vegetation:d_water** | -0.575 | 0.433 | -1.33 | 0.180 |  | -0.621 | 0.512 | -1.21 | 0.220 |  |
| **(Intercept)** | -0.370 | 0.844 | -0.44 | 0.660 | 157.8 | -0.537 | 0.676 | -0.79 | 0.427 | 157.2 |
| **TCD_sum** | 1.207 | 2.530 | 0.48 | 0.630 |  | 0.577 | 0.509 | 1.13 | 0.257 |  |
| **d_water** | -0.867 | 0.794 | -1.09 | 0.270 |  | -1.052 | 0.578 | -1.82 | 0.069 |  |
| **TCD_sum:d_water** | 0.680 | 1.986 | 0.34 | 0.730 |  | 0.179 | 0.536 | 0.33 | 0.739 |  |
| **(Intercept)** | -0.372 | 0.842 | -0.44 | 0.660 | 157.8 | -0.537 | 0.676 | -0.79 | 0.427 | 157.2 |
| **TCD_mean** | 1.200 | 2.517 | 0.48 | 0.630 |  | 0.577 | 0.509 | 1.13 | 0.257 |  |
| **d_water** | -0.868 | 0.792 | -1.10 | 0.270 |  | -1.052 | 0.578 | -1.82 | 0.069 |  |
| **TCD_mean:d_water** | 0.675 | 1.976 | 0.34 | 0.730 |  | 0.178 | 0.536 | 0.33 | 0.740 |  |
| **(Intercept)** | -0.778 | 0.698 | -1.12 | 0.265 | 155.7 | -0.696 | 0.701 | -0.99 | 0.321 | 157.0 |
| **Altitude** | 0.950 | 0.590 | 1.61 | 0.108 |  | 0.742 | 0.608 | 1.22 | 0.222 |  |
| **d_water** | -1.380 | 0.575 | -2.40 | **0.016** |  | -1.267 | 0.579 | -2.19 | **0.029** |  |
| **Altitude:d_water** | 1.136 | 0.817 | 1.39 | 0.164 |  | 0.834 | 0.875 | 0.95 | 0.341 |  |

Table S10 – GLMM results from multi-variable models of Total Bat Species, for 100m and 300m Buffers. R^2^ is only present in significant models. Bold *p-value*: significant values (*<0.05).

| **Total Species** | **Estimate** | **Std. Error** | **z value** | ***p-value*** | **R^2^ (fixed effects)** | **R2 (fixed effects+ random effects)** | **AIC** |
| --- | --- | --- | --- | --- | --- | --- | --- |
| **Buffer 100** | | | | | | | |
| **(Intercept)** | 0.631 | 0.137 | 4.61 | **4.0e-6** | 0.330 | 0.330 | 108.2 |
| **Altitude** | -0.167 | 0.147 | -1.14 | 0.254 |  |  |  |
| **d_water** | -0.369 | 0.147 | -2.51 | **0.012** |  |  |  |
| **Buffer 300** | | | | | | | |
| **(Intercept)** | 0.631 | 0.137 | 4.61 | **4.1e-06** | 0.330 | 0.330 | 108.1 |
| **Altitude** | -0.171 | 0.144 | -1.19 | 0.236 |  |  |  |
| **d_water** | -0.375 | 0.145 | -2.58 | **0.010** |  |  |  |

Table S11 – GLMM results from multi-variable Models of Total Bat Passes, for 100m and 300m Buffers. R^2^ is only present in significant models. Bold *p-value*: significant values (*<0.05).

| **Total Passes** | **Estimate** | **Std. Error** | **z value** | **Pr(>\|z\|)** | **R^2^ (fixed effects)** | **R2 (fixed effects+ random effects)** | **AIC** |
| --- | --- | --- | --- | --- | --- | --- | --- |
| **Buffer 100** | | | | | | | |
| **(Intercept)** | 2.126 | 0.311 | 6.84 | **7.8e-12** | 0.551 | 0.978 | 265.5 |
| **Urban** | 0.604 | 0.364 | 1.66 | 0.097 |  |  |  |
| **Altitude** | -0.040 | 0.362 | -0.11 | 0.912 |  |  |  |
| **d_water** | -0.852 | 0.373 | -2.28 | **0.023** |  |  |  |
| **Vineyard** | -0.516 | 0.309 | -1.67 | 0.094 |  |  |  |
| **d_water:Vineyard** | 0.845 | 0.459 | 1.84 | 0.066 |  |  |  |
| **Buffer 300** | | | | | | | |
| **(Intercept)** | 2.113 | 0.319 | 6.62 | **3.6e-11** | 0.516 | 0.978 | 267.9 |
| **Urban** | 0.298 | 0.396 | 0.75 | 0.451 |  |  |  |
| **Altitude** | -0.198 | 0.378 | -0.52 | 0.600 |  |  |  |
| **d_water** | -0.784 | 0.396 | -1.98 | **0.048** |  |  |  |
| **Vineyard** | -0.584 | 0.320 | -1.82 | 0.068 |  |  |  |
| **d_water:Vineyard** | 0.988 | 0.505 | 1.96 | 0.050 |  |  |  |

Table S12 – GLMM results from multi-variable models of *Pipistrellus kuhlii*, for 100m and 300m Buffers. R^2^ is only present in significant models. Bold *p-value*: significant values (*<0.05).

| ***P. kuhlii*** | **Estimate** | **Std. Error** | **z value** | **Pr(>\|z\|)** | **R^2^ (fixed effects)** | **R2 (fixed effects+ random effects)** | **AIC** |
| --- | --- | --- | --- | --- | --- | --- | --- |
| **Buffer 100** | | | | | | | |
| **(Intercept)** | 0.921 | 0.409 | 2.25 | **0.0245** | 0.681 | 0.958 | 208.0 |
| **Urban** | 0.718 | 0.400 | 1.80 | 0.0723 |  |  |  |
| **Vineyard** | -1.170 | 0.435 | -2.69 | **0.0071** |  |  |  |
| **Altitude** | -0.475 | 0.401 | -1.18 | 0.2365 |  |  |  |
| **d_water** | -1.560 | 0.385 | -4.05 | **5.1e-05** |  |  |  |
| **Buffer 300** | | | | | | | |
| **(Intercept)** | 0.156 | 1.004 | 0.16 | 0.877 | 0.840 | 0.977 | 211.8 |
| **Urban** | 0.023 | 0.466 | 0.05 | 0.960 |  |  |  |
| **Vineyard** | -1.117 | 0.482 | -2.32 | **0.021** |  |  |  |
| **Altitude** | -0.600 | 0.435 | -1.38 | 0.168 |  |  |  |
| **d_water** | -2.574 | 1.074 | -2.40 | **0.017** |  |  |  |
| **TCD_sum** | -2.537 | 2.415 | -1.05 | 0.293 |  |  |  |
| **d_water:TCD_sum** | -1.750 | 1.915 | -0.91 | 0.361 |  |  |  |

Table S13 – GLMM results from multi-variable Models of *Hypsugo savii*, for 100m and 300m Buffers. R^2^ is only present in significant models. Bold *p-value*: significant values (*<0.05).

| ***H. savii*** | **Estimate** | **Std. Error** | **z value** | **Pr(>\|z\|)** | **R^2^ (fixed effects)** | **R2 (fixed effects+ random effects)** | **AIC** |
| --- | --- | --- | --- | --- | --- | --- | --- |
| **Buffer 100** | | | | | | | |
| **(Intercept)** | -0.193 | 0.533 | -0.36 | 0.718 | 0.318 | 0.852 | 153.1 |
| **Mediterranean_vegetation** | 0.832 | 0.469 | 1.77 | 0.076 |  |  |  |
| **d_water** | -0.326 | 0.533 | -0.61 | 0.540 |  |  |  |
| **Vineyard** | -0.012 | 0.484 | -0.03 | 0.980 |  |  |  |
| **d_water:Vineyard** | 1.292 | 0.631 | 2.05 | **0.041** |  |  |  |
| **Buffer 300** | | | | | | | |
| **(Intercept)** | -0.415 | 0.613 | -0.68 | 0.499 | 0.263 | 0.874 | 154.5 |
| **Complex_cultivation_patterns** | -0.709 | 0.765 | -0.93 | 0.354 |  |  |  |
| **Mediterranean_vegetation** | 0.329 | 0.703 | 0.47 | 0.640 |  |  |  |
| **d_water** | -0.983 | 0.525 | -1.87 | 0.061 |  |  |  |
